# Plug-and-play – enzymatic amino acid production from methanol and carbon dioxide

**DOI:** 10.1038/s41467-026-74522-x

**Published:** 2026-06-17

**Authors:** Vivian Pascal Willers, Viktoria Lehmann, Susanne Müller, Ferdinand Vogelgsang, Arne Roth, Bastian Blombach, André Pick, Volker Sieber

**Affiliations:** 1 Chair of Chemistry of Biogenic Resources, Campus Straubing for Biotechnology and Sustainability, Technical University Munich, Straubing, Germany; 2 Professorship Microbial Biotechnology, Campus Straubing for Biotechnology and Sustainability, Technical University Munich, Straubing, Germany; 3 Fraunhofer Institute for Interfacial Engineering and Biotechnology IGB, Straubing, Germany; 4 SynBiofoundry@TUM, Technical University Munich, Straubing, Germany; 5 CASCAT GmbH, Europaring 4, Straubing, Germany; 6 School of Chemistry and Molecular Biosciences, The University of Queensland, Brisbane, QLD Australia

**Keywords:** Biocatalysis, Synthetic biology, Enzymes

## Abstract

Amino acids are the building blocks of proteins and thus among the macronutrients to feed humankind. They have extensive industrial applications as animal feed and food additives like dietary supplements, and flavor enhancers and moreover as chemical precursors. We present the design of a synthetic enzyme cascade system for synthesizing a series of amino acids from methanol. This acts as a case study on how to contribute to the sustainable, renewable energy-based supply of food and feed. The modular nature of the cascade allows for plug-and-play module swapping and fine tuning of enzyme composition to customize the target compound. The one-pot enzymatic systems, capable of utilizing methanol, ammonia, and, partially, carbon dioxide, were employed to synthesize glycine, serine, L-aspartic acid, L-valine, L-glutamic acid, and L-proline. Considering the increasing availability of methanol produced from carbon dioxide through thermocatalytic and even electrocatalytic and photocatalytic processes, methanol represents a key intermediate in future CO_2_-based value chains. In this context, our study provides a pathway for amino acid synthesis and food and feed production based on methanol and CO_2_ as carbon building blocks with reduced environmental impact.

## Abstract (web summary)

Amino acids have extensive applications as feed, food additives, and chemical precursors. Here the authors develop a modular enzymatic one-pot cascade system for amino acid synthesis from methanol and CO_2_.

## Introduction

Sustainable food production is becoming increasingly urgent as the global population continues to grow. Agriculture not only occupies large areas of land but also contributes approximately one-quarter of greenhouse gas emissions^1^, while overutilization of water resources threatens biodiversity and accelerates desertification. Meeting the rising demand for food requires innovative approaches that minimize environmental impact^2,3^.

Various strategies have been explored, ranging from algae-based food production and insect proteins to vertical farming. More recently, hybrid chemical–biological routes that combine heterogeneous catalysis and biocatalysis have enabled the direct synthesis of the three macronutrients, i.e., carbohydrates (in the form of starch), fats (in the form of triglycerides), and protein (in the form of amino acids) from carbon dioxide and hydrogen^4–7^. These approaches circumvent the low efficiency of plants in capturing light energy, being, for example, several orders of magnitude lower than state-of-the-art solar panels^8^. Despite these advances, such systems remain at an early stage (TRL ~ 4), and it requires more research and development effort for them to be implemented at a larger scale.

Among macronutrients, proteins, and specifically amino acids, offer a particularly promising target. Amino acids are already widely applied in animal feed, with millions of tons of lysine, methionine, valine, and others supplemented annually. In 2022, the amino acid market was valued at 25.6 billion USD and is expected to grow further^9^. However, current amino acid production is not necessarily more sustainable than sourcing proteins from plants cultivated on deforested land. Some amino acids, such as glycine, D, L-methionine, or L-aspartic acid, are still synthesized from fossil-based resources^10,11^, adding substantial quantities of carbon to the atmosphere^12,13^. While sugar fermentation can provide a more sustainable alternative (i.e., of biogenic resources), sugar production is limited by the low solar efficiency of plants, constraining yield per unit area and ultimately adding to the negative impact of agriculture on the environment. Developing methods to produce amino acids directly from CO₂ and renewable energy could therefore offer a more efficient and environmentally friendly solution.

CO_2_-derived C1 compounds, particularly methanol and formic acid, provide a versatile platform for chemical and biotechnological production. Methanol can be efficiently synthesized from CO_2_ via thermocatalytic hydrogenation^14^, and it serves as both a carbon and energy source^15–17^. In particular, methanol is especially promising as a substrate for biotechnological processes, combining the scalability and sustainability of CCU and Power-to-X approaches with the synthetic potential of biotechnology. Accordingly, methanol represents a valuable feedstock for amino acid production.

Natural and non-natural methylotrophic microorganisms are capable of utilizing methanol as the sole carbon source to synthesize diverse compounds^18,19^. *Bacillus methanolicus* is one such organism, capable of producing up to 59 g l^−1^ L-glutamic acid using methanol, with a yield of 0.19 g L-glutamic acid-carbon/ g methanol-carbon^20^. Nevertheless, the feasibility of methylotrophic organisms for industrial-scale applications remains limited^21^, and methanol and formaldehyde toxicity must be overcome to increase product titers^22^.

A complementary or alternative approach involves cell-free enzymatic cascades, which can convert methanol and CO₂ directly into a variety of chemicals, fuels, and nutrients^23,24^. For example, Cai et al. developed a three-module enzymatic system to produce starch from CO₂^4^. CO₂ was first converted chemically into methanol, which was then gradually converted into the C3 compound dihydroxyacetone, serving as a precursor for starch. Similar three-module systems have been applied to produce polyhydroxybutyric acid (PHB) from CO₂^25^, involving chemical conversion of CO₂ to methanol, condensation to glycolaldehyde, and subsequent synthesis of PHB via acetyl-CoA^5^. The acetyl-CoA platform also enables the production of fatty acids, which are major constituents of fats and oils^26^. Importantly, the same principle of modular enzyme cascades can be extended to the synthesis of amino acids, allowing the construction of both small and larger amino acids from methanol and CO₂. Indeed, recent studies have demonstrated the production of small amino acids, such as L-alanine, glycine, and serine, directly from methanol or CO₂ in enzymatic or chemo-enzymatic cascade systems, achieving carbon yields of 30–90%^6,27,28^.

In this study, we expand the concept of amino acid production from carbon dioxide and hydrogen to include a broader range of amino acids. To achieve this, we exploit the flexibility of cell-free enzyme cascades, leveraging the potential of CO₂ as a carbon source through a combination of promiscuous and selective enzyme reactions to synthesize amino acids from methanol and CO₂ (Supplementary Table 1). The interchangeability of the enzyme system creates a highly versatile toolbox, enabling the production of a wide variety of amino acids, from small, two-carbon compounds, such as glycine, to larger, partially essential amino acids with up to five carbon atoms, including L-glutamic acid and L-valine.

## Results

### Design and optimization of enzyme cascades for the production of amino acids from methanol

**Fig. 1:**
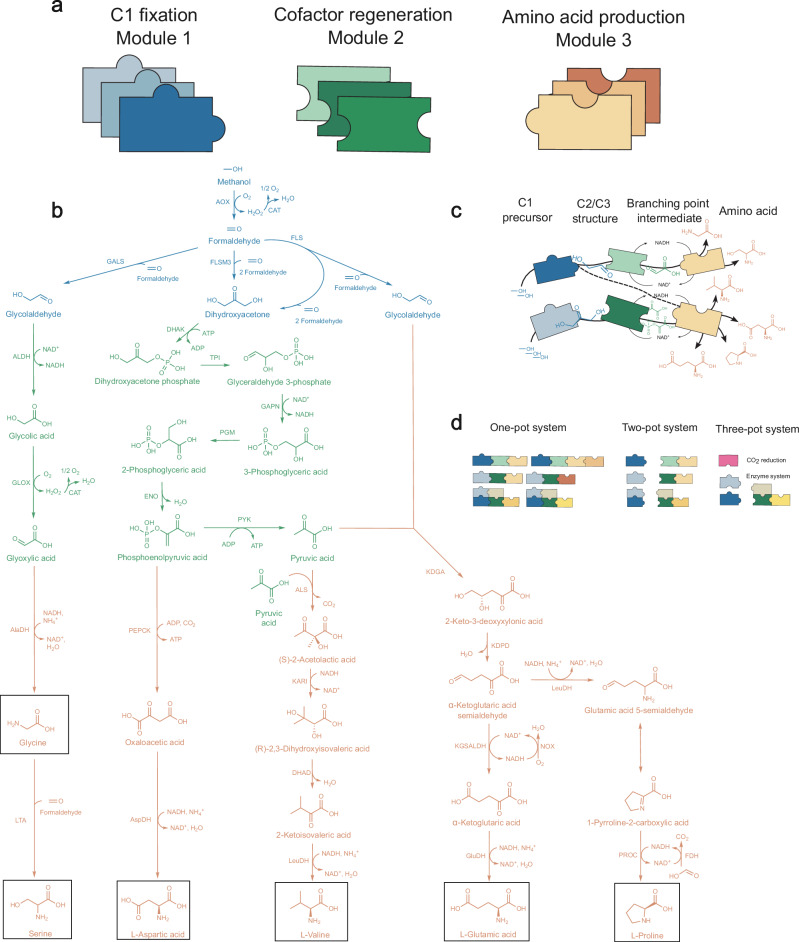
Enzymatic plug-and-play system for the production of diverse amino acids. **a** Modular architecture with three major components: Methanol-dependent C1 fixation (module 1) in blue. Cofactor regeneration (module 2/core module) in green. Amino acid synthesis (module 3) in orange. **b** Proposed pathways of enzymatic amino acid production based on methanol and CO_2_ with an intrinsic cofactor regeneration system. Methanol is converted via alcohol oxidase (AOX) to formaldehyde, which is combined to glycolaldehyde, dihydroxyacetone, or a mixture of both via glycolaldehyde synthase (GALS), formolase M3 (FLSM3), or formolase “wildtype” (FLS), respectively. For glycine and serine, glycolaldehyde is oxidized via aldehyde dehydrogenase (ALDH) and glycolate oxidase (GLOX) to produce glyoxylic acid, from which glycine is obtained by L-alanine dehydrogenase (AlaDH). Glycine is further converted to serine via a low-specific-activity threonine aldolase (LTA). For the four C4 and C5 amino acids, dihydroxyacetone is phosphorylated by dihydroxyacetone kinase (DHAK) and isomerized by triosephosphate isomerase (TPI). Glyceraldehyde 3-phosphate is oxidized by glyceraldehyde 3-phosphate dehydrogenase (GAPN). 3-Phosphoglyceric acid is isomerized by phosphoglycerate mutase (PGM) and dehydrated by enolase (ENO). For L-aspartic acid synthesis, phosphoenolpyruvic acid is carboxylated and dephosphorylated to oxaloacetic acid by phosphoenolpyruvic acid carboxykinase (PEPCK), followed by reductive amination by L-aspartate dehydrogenase (AspDH). Alternatively, phosphoenolpyruvic acid is converted to pyruvic acid via pyruvate kinase (PYK). To produce L-valine, two pyruvic acid molecules are condensed by acetolactate synthase (ALS), reduced and rearranged by ketol-acid reductoisomerase (KARI), and dehydrated by dihydroxy acid dehydratase (DHAD). L-Leucine dehydrogenase (LeuDH) catalyzes the reductive amination of 2-ketoisovaleric acid to produce L-valine. For L-glutamic acid and L-proline, pyruvic acid and glycolaldehyde are condensed by 2-dehydro-3-deoxy-D-gluconate aldolase (KDGA) and converted to α-ketoglutaric acid semialdehyde by 2-keto-3-deoxy-D-xylonate dehydratase (D-KDPD). This branching point is either oxidized by α-ketoglutaric acid semialdehyde dehydrogenase (KGSALDH) or reductively aminated by L-leucine dehydrogenase (LeuDH). α-Ketoglutaric acid is reductively aminated to L-glutamic acid by L-glutamate dehydrogenase (GluDH). Contrarily, glutamic acid semialdehyde spontaneously cyclizes and is then reduced to L-proline by pyrroline-5-carboxylate reductase (PROC). To maintain cofactor balance, NADH oxidase (NOX) and formate dehydrogenase (FDH) are included in the L-glutamic acid and L-proline cascade, respectively. **c** Plug-and-play concept used in this study to diversify the cascade output. **d** The modular design allows for a one-, two-, or three-pot system.

To develop an enzymatic plug-and-play system for producing diverse amino acids, we designed a modular architecture with three core components (Fig. 1a): methanol-dependent C1 fixation (module 1), cofactor regeneration (module 2/core module), and amino acid synthesis (module 3). For that, the previously published enzymatic production of L-alanine from methanol served as a promising starting point^6^.

In module 1, methanol is oxidized by an alcohol oxidase and condensed via a formaldehyde condensing enzyme^29^, yielding dihydroxyacetone (C3) and glycolaldehyde (C2) as key precursors (Fig. 1b). The formaldehyde condensation step is pivotal, as it determines the ratio of C2/C3 precursors and thus the spectrum of producible amino acids (Fig. 1c)^30^. The formolase (FLS) variant FLSM3^4^ was employed to redirect the output of module 1 to dihydroxyacetone, taking advantage of the developed formaldehyde condensing enzymes. Conversely, replacing FLSM3 with glycolaldehyde synthase (GALS)^5^ would result in a shift in the product outcome towards glycolaldehyde. Finally, the use of the “wildtype” of FLS^29^ was considered, which would result in a mixture of glycolaldehyde and dihydroxyacetone as the product. Different FLS variants were assessed previously in terms of kinetics and product preference^4,5,30^. As a compromise, these data suggested FLS to be less specific in product outcome and could serve as a starting point to produce both precursor molecules (C2 and C3) simultaneously.

Dihydroxyacetone functions as a bridging molecule within the system, thereby enabling the redirection of carbon flux into the semi-phosphorylated Entner–Doudoroff (ED) pathway (module 2), to facilitate the production of phosphoenolpyruvic acid, the central metabolite pyruvic acid, and NADH. Glycolaldehyde similarly enters the glycolaldehyde oxidation pathway to form glyoxylic acid.

The amino acid production module (module 3) converts pyruvic acid or glyoxylic acid into target amino acids, using pathway-specific enzymes and a final amination step catalyzed by promiscuous amino acid dehydrogenases, requiring NADH and ammonia.

**Fig. 2:**
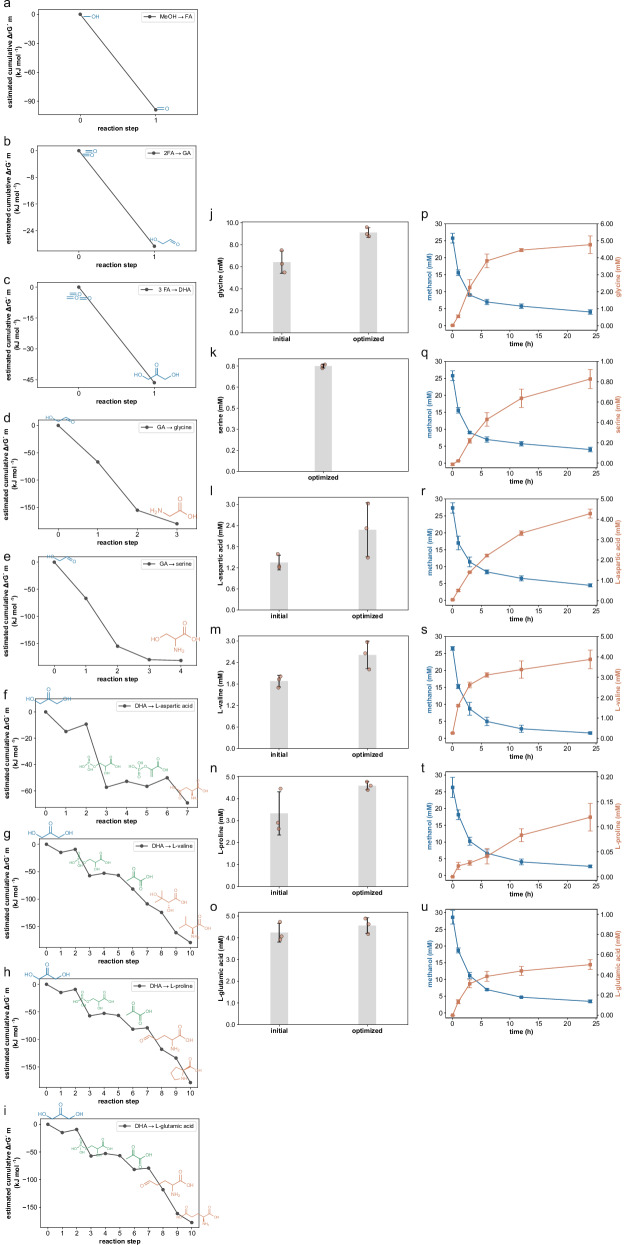
Design and optimization of enzyme cascades for the production of amino acids from methanol. **a–i** Estimation of reaction Gibbs energy for 1 mM as standard concentration of enzymatic amino acid system by using eQuilibrator 3.0 web tool in default mode. Glycine and serine cascade starting reaction is the oxidation of glycolaldehyde, starting reaction of all other cascades is the phosphorylation of dihydroxyacetone. **a** Methanol oxidation to formaldehyde. **b** Condensation of formaldehyde to glycolaldehyde. **c** Condensation of formaldehyde to dihydroxyacetone. **d** Conversion of glycolaldehyde to glycine. **e** Conversion of glycolaldehyde to serine. **f** Conversion of dihydroxyacetone to L-aspartic acid. **g** Conversion of dihydroxyacetone to L-valine. **h** Conversion of dihydroxyacetone to L-proline. **i** Conversion of dihydroxyacetone to L-glutamic acid. **j–o** Combination of amino acid synthesis and cofactor regeneration module. To optimize amino acid production, shortened pathways with the starting molecules dihydroxyacetone and glycolaldehyde were used. The amino acid yield after 5 h of reaction time is shown before (initial) and after optimization (optimized). **j** Glycine. **k** Serine. **l** L-Aspartic acid. **m** L-Valine. **n** L-Proline. The yield of L-proline was measured after 7 h of reaction time. **o** L-Glutamic acid. Data are presented as mean values with error bars representing the standard deviation of triplicates (*n* = 3 samples). Individual data points are shown using overlaid dot plots. **p**–**u** Combination of amino acid synthesis, cofactor regeneration and C1-fixation module. Time curve of methanol consumption in blue and amino acid production in orange. **p** Glycine. **q** Serine. **r** L-Aspartic acid. **s** L-Valine. **t** L-Proline. **u** L-Glutamic acid. Data are presented as mean values with error bars indicating the standard deviation of triplicates (*n* = 3 samples). At 0 h, signals for serine, L-proline, and L-glutamic acid were negligible and were thus set to 0 mM. Abbreviations: MeOH, methanol. FA, formaldehyde. GA, glycolaldehyde. DHA, dihydroxyacetone. Source data for this figure are provided as a Source Data file.

Having this modular system (Fig. 1d) in mind, we drafted possible routes from pyruvic acid or intermediates of the cascades for the amino acids glycine as a C2-amino acid, serine as a C3-amino acid, L-aspartic acid as a C4-amino acid, L-valine, L-glutamic acid, and L-proline as C5-amino acids. To design possible pathways, we used the KEGG^31^ and BioCyc^32^ databases. Furthermore, we gathered information on enzyme availability, kinetics, and properties from BRENDA^33^. To evaluate the formation of the target product in the pathways, we determined the thermodynamics of pathway reactions via the eQuilibrator 3.0 web tool^34^ (Fig. 2a–i, Supplementary Table 2).

### Determining the final amination step

To minimize complexity in the amino acid synthesis module, we reverse-engineered pathways to reduce enzyme variability, ideally relying on a single promiscuous amino acid dehydrogenase. The amino acid dehydrogenase catalyzes the final amination step, converting precursor molecules into target amino acids. We began with a promiscuous L-alanine dehydrogenase (*bs*AlaDH)^35^, which, despite its primary activity on pyruvic acid^6^, demonstrated promising kinetics on glyoxylic acid for glycine synthesis (Supplementary Table 3), as well as activity on 2-ketoisovaleric acid and hydroxypyruvic acid (Supplementary Fig. 1), precursors for L-valine and serine, respectively. As we can implement it in the glycine pathway, however, the significant difference in activity between pyruvic acid and 2-ketoisovaleric acid necessitated the use of a more specific leucine dehydrogenase (*bs*LeuDH) for efficient L-valine synthesis (Supplementary Table 3). As with *bs*AlaDH, carbon would be redirected to L-alanine instead of L-valine. Remarkably, *bs*LeuDH exhibited broad promiscuity, including activity on α-ketoglutaric acid semialdehyde (Supplementary Table 3), enabling a novel pathway for L-proline synthesis, and even slight activity on 2-keto-4-hydroxybutyric acid, suggesting potential for L-lysine production (Supplementary Fig. 1).

For the synthesis of the amino acids L-aspartic acid and L-glutamic acid, we employed a bacterial aspartate dehydrogenase (*vp*AspDH), identified through genome mining, which showed high activity on oxaloacetic acid for L-aspartic acid production (Supplementary Table 3). However, its negligible activity on α-ketoglutaric acid (Supplementary Fig. 1) led us to adopt a commercial L-glutamate dehydrogenase from *Bos taurus* for L-glutamic acid synthesis (Supplementary Table 4).

### Determining the enzymes for the amino acid synthesis module

With the amination step established, we next focused on connecting it to the production of precursor molecules from either pyruvic acid or intermediates of the semi-phosphorylated ED pathway (Supplementary Note 1). For L-valine, L-glutamic acid, and L-proline, which derive from pyruvic acid, we prioritized robust enzymes compatible with the core module’s conditions (HEPES pH 7.5, 30 °C). The L-valine pathway, branching from pyruvic acid, utilized homologs of acetolactate synthase (*bs*ALS), ketol-acid reductoisomerase (*gs*KARI), and dihydroxy-acid dehydratase (*st*DHAD), all of which demonstrated suitable activity and stability under these conditions^23^.

The pathways for L-proline and L-glutamic acid share the initial enzymes 2-dehydro-3-deoxy-D-gluconate aldolase (*pt*KDGA) and 2-keto-3-deoxy-D-xylonate dehydratase (*pp*D-KDPD), which convert intermediates into α-ketoglutaric acid semialdehyde. We chose two enzymes that showed promising activity at pH 7.5 (Supplementary Table 5). Furthermore, both enzymes showed good compatibility with our system, with robust temperature stability^36,37^. From this point, the pathways diverge: for L-proline, we chose *bs*LeuDH as it can catalyze the key amination step of α-ketoglutaric acid semialdehyde, while for L-glutamic acid, *Pseudomonas putida* α-ketoglutaric acid semialdehyde dehydrogenase (*pp*KGSALDH) oxidizes the semialdehyde to α-ketoglutaric acid by using NAD^+^ as a cofactor^38^. This cofactor preference is desired for our cofactor recycling system. Due to that *pp*KGSALDH is an ideal candidate for integration into the system. Similar to that, cofactor compatibility was the major consideration for the integration of the pyrroline-5-carboxylate reductase (*ec*PROC)^39^ in catalyzing the final step in the L-proline pathway using NADH.

For L-aspartic acid synthesis, the core cascade was shortened by replacing pyruvate kinase with a phosphoenolpyruvic acid carboxykinase from *Escherichia coli* (*ec*PEPCK), which recycles ATP and uses ammonium bicarbonate for carboxylation, maintaining cofactor balance and compatibility with the core module. In addition to that, in cascade conditions, it shows excellent activity on phosphoenolpyruvic acid (Supplementary Table 3). Furthermore, ammonium bicarbonate as a substrate serves both, as CO_2_ source and as an ammonium source in the cascade. In contrast to recently reported rGlyP-based aspartic acid synthesis^40^, this route offers a more direct and seamlessly integrable module for our system without requiring the complexity of the reductive glycine pathway.

Glycine synthesis presented a unique challenge, as its two-carbon structure made its production from dihydroxyacetone uneconomical. Instead, we used glycolaldehyde as a substrate. By evaluating the results of the database search, it is notable that, in contrast to previously published work^27,28^, glycolaldehyde provides direct access to glycine and serine with reduced pathway complexity. However, for the envisioned system, a double oxidation to generate glyoxylic acid is required. To avoid unwanted oxidation of formaldehyde, we selected an engineered *Thermoplasma acidophilum* aldehyde dehydrogenase (M42)^41–43^, which was originally used for the specific oxidation of glyceraldehyde. By testing its promiscuity, we observed activity toward glycolaldehyde but barely any activity towards formaldehyde (Supplementary Table 3). Glyoxylic acid production was achieved using spinach glycolate oxidase, which is one of the few characterized glycolate oxidases expressible in *Escherichia coli*. The pathway was completed by incorporating a low-specificity threonine aldolase (LTA)^44^ to enable glycine-to-serine conversion.

### Building a modular enzyme cascade: Combination of amino acid synthesis module and core module

After we set the amino acid synthesis module, we combined the amino acid synthesis modules with the core module to test the plug-and-play system (Fig. 2j-o). For glycine and serine pathways, we used the shortened core module as described earlier. The amino acid synthesis module was normalized to 0.9 U mL^-1^, while core module enzyme concentrations followed the previously reported conditions^6^ (Supplementary Table 6, Supplementary Table 7). This setup allowed the production of all target amino acids (Table 1). Initial conversions achieved glycine synthesis with 65% conversion from glycolaldehyde, the highest efficiency observed. For dihydroxyacetone-derived pathways, L-valine reached 36% conversion, and the dual-substrate system for L-glutamic acid achieved 42% conversion. To improve efficiency, we adjusted key enzyme concentrations, which play a role in cofactor recycling and might, if the cofactor is not regenerated quickly enough, be a bottleneck for the whole flux of the system (Supplementary Table 7). Increasing L-alanine dehydrogenase fivefold raised glycine conversion to 91% (Fig. 2j), whereas comparable systems have reported yields of 86%^45^. Elevating *gs*KARI concentration improved L-valine conversion to 52% (Fig. 2m). In the dual-substrate cascade, *pt*KDGA showed a low specificity toward glycolaldehyde, so we assumed that an increase in *pt*KDGA levels enhances the aldol condensation reaction to form 2-keto-3-deoxyxylonic acid. However, increasing *pt*KDGA enhanced L-glutamic acid conversion only slightly to 46% (Fig. 2o). For enhanced L-aspartic acid synthesis, together with the key enzyme *ec*PEPCK, we also increased the ammonium bicarbonate concentration to counter the high K_m_ value of *ec*PEPCK for carboxylation. This raised the conversion to 23% from dihydroxyacetone (Fig. 2l).

**Table 1:** Comparison of amino acid production cascades using different starting substrates

Substrate	Substrate (mM)	System	Reaction time (h)	Final volume (mL)	Target product	Final titer (mM)	*Carbon yield (%)
Glycolaldehyde	10	One-pot	5	0.2	Glycine	9.1 ± 0.4	91
Glycolaldehyde +Formaldehyde	10	One-pot	5	0.2	Serine	0.8 ± 0.0	8
Dihydroxyacetone + CO_2_	10	One-pot	5	0.2	L-Aspartic acid	2.3 ± 0.8	23
Dihydroxyacetone	10	One-pot	5	0.2	L-Valine	2.6 ± 0.4	52
Glycolaldehyde + Dihydroxyacetone	10	One-pot	5	0.2	L-Glutamic acid	4.6 ± 0.4	46
Glycolaldehyde + Dihydroxyacetone	10	One-pot	7	0.2	L-Proline	4.6 ± 0.2	46
Methanol	30	One-pot	24	0.2	Glycine	4.8 ± 0.5	32
Methanol	30	One-pot	24	0.2	Serine	0.8 ± 0.1	8
Methanol	30	One-pot	24	0.2	L-Aspartic acid	4.3 ± 0.2	43
Methanol	30	One-pot	24	0.2	L-Valine	3.9 ± 0.5	78
Methanol	30	One-pot	24	0.2	L-Glutamic acid	0.5 ± 0.1	8
Methanol	30	One-pot	24	0.2	L-Proline	0.1 ± 0.0	2
CO_2_ via Methanol	300 (150)**	Two-pot three-steps	50	1.0***	L-Valine	11.1 ± 0.6	44
CO_2_ via Methanol	60 (30)**	Two-pot three-steps	45	15.0***	L-Aspartic acid	4.0	40

* Carbon yield calculated based on methanol-derived carbon. CO_2_ release was not considered in the calculation.

** Theoretical starting concentration of methanol after diluting pot one with pot two.

*** Final volume of reaction after addition of pot two to pot one.

Product titers are shown of optimized cascades. For the two-pot systems, initial substrate concentration of the first pot is shown. In brackets, the theoretical substrate concentration based on the one-to-one dilution of the first pot with the second pot is shown. Data are presented as mean values with errors indicating the standard deviation of triplicates (*n* = 3 samples). Source data for this table are provided as a Source Data file.

### Building a pathway: Combination of amino acid synthesis, core, and C1 module

With the amino acid synthesis and core modules optimized, we integrated the C1 module to complete the plug-and-play system. The C1 module, consisting of methanol oxidase and a formaldehyde condensing enzyme, was designed to be flexible, allowing for the incorporation of different enzyme variants. We used three distinct variants, two formolase variants and one glycolaldehyde synthase, to maximize adaptability and tailor the production of precursor molecules for specific amino acid targets.

For glycine and serine synthesis, methanol oxidase and glycolaldehyde synthase (GALS) formed module 1. GALS directed formaldehyde predominantly toward glycolaldehyde production, avoiding dihydroxyacetone formation. Combining the C1-fixation module (Supplementary Table 8) with the glycine system (Supplementary Table 6) enabled the synthesis of 4.8 mM glycine from 30 mM methanol within 24 hours (Fig. 2p). Incorporating LTA produced 0.8 mM serine from methanol alone (Fig. 2q), with 3.5 mM glycine remaining, likely due to the low specificity of threonine aldolase. Importantly, this cascade synthesized both glycine and serine entirely from methanol, demonstrating dual formaldehyde fixation in a single system.

To produce amino acids with longer carbon chains, we replaced GALS with FLSM3, shifting module 1 output toward dihydroxyacetone (Supplementary Table 8). Integrating this C1-fixation module with the L-aspartic acid and L-valine systems (Supplementary Table 7) converted 30 mM methanol into 4.3 mM L-aspartic acid and 3.9 mM L-valine within 24 hours (Fig. 2r, s). Surprisingly, conversions exceeded those starting from dihydroxyacetone. A possible reason for this increase in performance could be dihydroxyacetone reactivity. Dihydroxyacetone is a reactive compound. Its carbonyl group can react with amines that are present at high concentrations in the reaction mixture (e.g., free ammonium, protein residues, or buffer components)^46^. We assume that the switch to methanol leads to lower transient dihydroxyacetone concentrations, which can be immediately converted by dihydroxyacetone kinase due to its very low K_m_ and high activity. With this direct flux, elevated concentrations are diminished, resulting in reduced side product formation.

The final challenge involved producing the C5 amino acids L-glutamic acid and L-proline, which required the simultaneous generation of both glycolaldehyde and dihydroxyacetone. We utilized the “wildtype” formolase (FLS, Supplementary Table 8), which generates a mixture of these precursors, though not in an ideal 1:1 ratio. Despite this limitation, the system successfully produced 0.1 mM L-proline and 0.5 mM L-glutamic acid from 30 mM methanol (Fig. 2t, u). Notably, in our pathway design, both these C5 amino acids are derived from methanol without any carbon loss, demonstrating the versatility and potential of the plug-and-play approach. However, an imbalance in the system was evident: L-alanine, a side product, accumulated to 0.8 mM during L-glutamic acid synthesis and 1.0 mM during L-proline synthesis after 24 h. This byproduct formation likely stems from the non-ideal ratio of glycolaldehyde to dihydroxyacetone. When dihydroxyacetone is produced in excess, the transient concentration of glycolaldehyde decreases. Consequently, *pt*KDGA, the critical enzyme for L-proline and L-glutamic acid formation, exhibits reduced activity due to its low specificity for glycolaldehyde. Since NAD^+^ is recycled during L-alanine formation, the cascade continues to favor the side product rather than the desired L-glutamic acid or L-proline. Notably, the high concentration of L-alanine in the L-proline synthesis pathway may be attributed to the side activity of *bs*LeuDH, as its K_m_ for pyruvic acid is approximately one order of magnitude lower than its K_m_ toward α-ketoglutaric acid semialdehyde (Supplementary Table 3).

### Amino acid production from CO_2_–based methanol and ammonia

The flexibility and robustness of our enzyme system were demonstrated by synthesizing six different amino acids from methanol, ranging from the small C2-carbon amino acid glycine to C5-carbon amino acids, such as L-valine or L-glutamic acid. To further validate the entire production chain, we targeted L-valine and L-aspartic acid production by directly starting from CO_2_ as the primary carbon source. Furthermore, we explored the effects of preliminary scaling in substrate load and reaction volume on system performance.

CO_2_ was used to synthesize methanol through thermocatalytic hydrogenation. Conventionally, coke-derived synthesis gas (a mixture of CO, CO_2_, and H_2_ of defined concentrations) undergoes a thermocatalytic treatment to form methanol and water. This reaction mixture is distilled to obtain highly pure methanol. In the future, synthesis gas mixtures of only CO_2_ and H_2_ (without carbon monoxide) are likely to play a more prominent role as feedstock for methanol synthesis, since CO_2_ is considered an important future carbon source for renewable value chains. With our experiment, we want to demonstrate that our developed enzymatic cascade for synthesizing amino acids (here, L-valine and L-aspartic acid) also shows a promising performance when applying CO_2_-derived methanol. Additionally, we forgo the purification step of the methanol / H_2_O mixture - instead, the raw product mixture as obtained after the reactor was fed into the enzymatic cascade.

The synthesis of methanol from CO_2_ is proceeding via different C1 intermediates, including CO.

These reactions are heterogeneously catalyzed at elevated temperatures and pressures with the use of CuZnAlO_x_ (CZA) catalysts. For this study, methanol was synthesized in a fixed-bed reactor system, which is described in more detail in the methods section. The reaction was carried out at steady-state conditions at 40 bar total pressure and 230 °C reaction temperature. The molar ratio of H_2_ / CO_2_ was adjusted to 3, and the weight hourly space velocity (WHSV = 0.2 – 3.9 h^-1^) was changed by varying the total volumetric flow of the gases between 30 and 90 mL min^-1^ at catalysts loadings (industrial CZA) of 21.5 mg and 194.0 mg, respectively.

At space velocities below 0.5 h^-1^ no change in total CO_2_ conversion (X_CO2_ = 17 mol-%) and CH_3_OH yield (Y_CH3OH_ = 11 mol-%) was observed, which indicates that the reaction was at chemical equilibrium. At this point, methanol was produced at a production rate of 1.55 g_CH3OH_ g_cat_^-1^ h^-1^ (WHSV = 0.44 h^-1^). Note that no other carbon-containing product than CO and CH_3_OH was detected. All liquid samples collected during the experiment on WHSV variation were unified and sent for HPLC analysis. The final concentration of methanol in water was 17%.

**Fig. 3:**
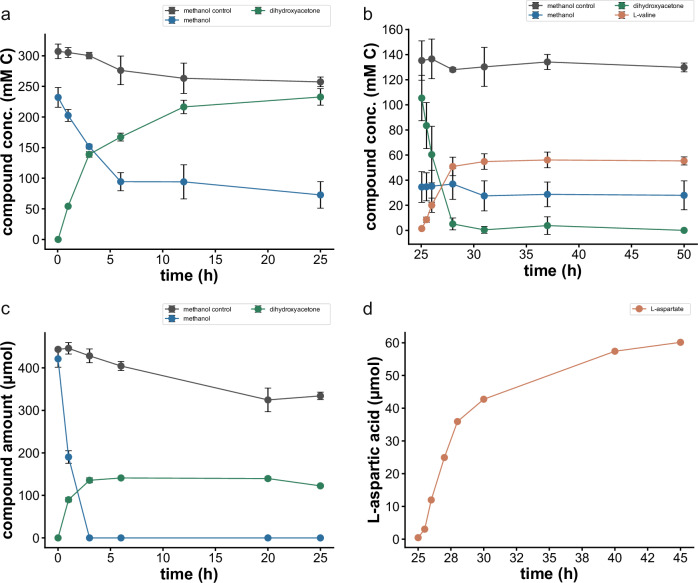
Two-step approach of L-valine and L-aspartic acid synthesis from CO_2_-derived methanol. L-Valine system for substrate scaling starting from 300 mM methanol in a final volume of 1 mL. L-Aspartic acid system for volume scaling starting with 60 mM methanol in a final volume of 15 mL. For the L-valine system, 500 µL of the first step after 25 h were added to 500 µL of a freshly prepared enzyme and cofactor mixture for step two. Because of that, concentrations of step two are diluted by a factor of two. Similar to that, 7.5 mL of step one in the L-aspartic acid synthesis cascade was added to 7.5 mL of an enzyme and cofactor mixture for step two. **a**, **b** L-Valine synthesis. **a** The first step. Dihydroxyacetone synthesis in green from CO_2_-derived methanol in blue. Data are presented as mean values with error bars indicating the standard deviation of triplicates (*n* = 3 samples). Y-axis in mM carbon (mM C). At 0 h, the dihydroxyacetone signal was negligible and was thus set to 0 mM C. **b** The second step. L-Valine synthesis in orange from dihydroxyacetone in green. In gray, a control experiment without the addition of methanol oxidase is shown. Data are presented as mean values with error bars indicating the standard deviation of triplicates (*n* = 3 samples). Y-axis in mM carbon (mM C). At 50 h, the dihydroxyacetone signal was negligible and was thus set to 0 mM C. **c**, **d** L-Aspartic acid synthesis. **c** The first step. Dihydroxyacetone synthesis in green from CO_2_-derived methanol in blue. In gray, a control experiment without the addition of methanol oxidase is shown. Data are presented as mean values with error bars generally indicating the standard deviation of triplicates (*n* = 3 samples), except for the time points 3 h, 6 h, and 20 h of the dihydroxyacetone quantification, where they represent the standard deviation of duplicates (*n* = 2 samples). Y-axis as amount (µmol). At 3 h, 6 h, 20 h, and 25 h, methanol signals were negligible and were thus set to 0 µmol. Likewise, at 0 h, the dihydroxyacetone signal was negligible and was thus set to 0 µmol. **d** The second step. L-Aspartic acid synthesis in orange from dihydroxyacetone. L-Aspartic acid measurement was conducted once (*n* = 1 sample). Y-axis as amount (µmol). Source data for this figure are provided as a Source Data file.

To demonstrate scalability in substrate amount, we converted a 300 mM CO₂-based methanol solution to L-valine. Given the high K_m_ and relatively low activity of formolase for formaldehyde formation, we divided the biocatalytic process into two steps. First, methanol was converted to dihydroxyacetone using methanol oxidase, catalase, and FLSM3. This allowed us to increase alcohol oxidase concentration while reducing FLSM3’s concentration, as higher formaldehyde titers enhanced FLSM3 activity without affecting other sensitive enzymes in the cascade. In the first step, 300 mM methanol was converted to 78 mM dihydroxyacetone (234 mM carbon) within 25 hours in a 500 µL volume, achieving a 78% carbon yield (Fig. 3a). For the second step, enzymes from module 2 and module 3 were added, resulting in a one-to-one dilution of the reaction mixture to a final volume of 1 mL. This dilution reduced the dihydroxyacetone concentration from 78 mM to 39 mM (117 mM carbon). Dihydroxyacetone was then converted to L-valine within additional 25 h, yielding 11 mM L-valine. L-valine synthesis stopped after 12 h, respectively. Given the stoichiometry (2 dihydroxyacetone → 1 L-valine + 1 CO₂), this corresponds to a 56% molar conversion efficiency of dihydroxyacetone to L-valine and a 47% carbon conversion efficiency. The overall carbon yield, accounting for CO₂ release, was 37% (44% without taking the CO_2_ release into account; Fig. 3b). This result underscores the potential of the cascade system, as the conditions and enzyme concentrations from previous experiments were maintained while achieving a fivefold increase in substrate concentration and reaction volume. This scaling effort resulted in a sevenfold increase in theoretical yield compared to the initial 200 µL proof-of-principle cascade. However, L-alanine was identified as the major side product, reaching concentrations of up to 4 mM (Supplementary Fig. 2). Despite the substrate preference of *bs*LeuDH, which favors the L-valine precursor 2-ketoisovaleric acid over pyruvic acid, apparently a residual activity on pyruvic acid likely contributed to the formation of L-alanine. When including L-alanine as a potential target compound, overall carbon yield, accounting for CO₂ release on total amino acid production, would rise to 45%.

Further analysis of the samples using LC-MS aimed to identify potential intermediate accumulation and bottleneck reactions. The metabolite profile indicated that intermediates of the L-valine module were predominantly present in the system. 2,3-Dihydroxyisovaleric acid accumulated rapidly within the first hour but declined shortly thereafter. Phosphoenolpyruvic acid was detected initially but was no longer visible after three hours. Transient increases in pyruvic acid and 2-ketoisovaleric acid concentrations were also observed (Supplementary Fig. 2). Despite these fluctuations, no significant accumulation of pathway intermediates was detected, suggesting that the missing carbon may have been converted into unidentified byproducts. In the future, optimization of L-valine pathway enzymes is important to increase L-valine yield and lower L-alanine concentration as a side product. By creating enzymes with increased activity, flux from pyruvic acid to 2-ketoisovaleric acid could be enhanced to diminish L-alanine production. A possible first target here could be *gs*KARI, as we needed to increase its concentration due to low activity, but also *st*DHAD, as its substrate, showed the highest transient concentration, which probably led to a slowed L-valine production.

The initial proof-of-principle cascade for L-aspartic acid production achieved a conversion efficiency of 43%, yielding 4.3 mM L-aspartic acid from 30 mM methanol and ammonium bicarbonate. While this system benefits from double CO₂ fixation, it was initially tested at a small scale of 200 µL. Scaling the substrate concentration would require adjustments to ammonium bicarbonate levels, complicating the system without further enzyme engineering. Therefore, we focused on scaling the reaction volume while maintaining similar substrate loads. To demonstrate the potential for volume expansion, the system was scaled from 200 µL to 15 mL, representing a 75-fold increase. Similar to the L-valine system, the C1 module was separated from module 2 and module 3 to avoid interference from elevated concentrations of formaldehyde and methanol. After 6 h reaction time, the initial 60 mM methanol was converted to 19 mM dihydroxyacetone (Fig. 3c). To align with the timeline of the L-valine system, modules 2 and 3, along with the necessary cofactors and additives, were added after 25 hours, during which dihydroxyacetone concentrations remained mostly stable. This resulted in a one-to-one dilution of the system, with 7.5 mL of the reaction mixture combined with 7.5 mL of modules 2 and 3, achieving a final volume of 15 mL. Upon the addition of these modules, L-aspartic acid formation commenced immediately, reaching a concentration of 4.0 mM after 20 h (Fig. 3d). This result is comparable to the 4.3 mM achieved in the 200 µL scale, demonstrating excellent scalability. Due to the increased volume, the theoretical yield of L-aspartic acid rose from 0.9 to 60.0 µmol.

Further analysis of the samples using LC-MS revealed that phosphoenolpyruvic acid and 2-phosphoglyceric acid or 3-phosphoglyceric acid accumulated during the first two hours of the cascade but decreased after five hours and were no longer detectable thereafter. Slight traces of pyruvic acid were also observed, potentially due to minor side activity of *ec*PEPCK. However, no major side products or bottlenecks were identified, as cascade components were maintained at low concentrations (Supplementary Table 9). The missing carbon is therefore likely attributable to yet unidentified side products. Currently, one major bottleneck in the cascade is the low specificity of *ec*PEPCK on bicarbonate, which needs to be tackled in the future.

## Discussion

The development of a modular enzymatic cascade capable of synthesizing six structurally distinct amino acids, ranging from two to five carbons, directly from CO₂-derived methanol marks a fundamental advance in sustainable biocatalysis. In initial small-scale proof-of-principle cascades, 91% glycine yield from glycolaldehyde in a cofactor-neutral system, up to 78% L-valine conversion from 30 mM methanol, methanol utilization coupled to direct CO₂ fixation with 40% L-aspartic acid yield, and a direct route from methanol to the amino acids L-proline and L-glutamic acid, this work demonstrates the feasibility of a circular carbon economy for amino acid production. These results not only validate the technical robustness of the modular approach but also highlight its potential to reshape amino acid supply chains by decoupling amino acid synthesis from traditional agricultural or petrochemical inputs.

A defining feature of this modular system is, besides its flexibility in amino acid outcome, a second level of variability by unplugging module 1 from module 2 and module 3 to circumvent elevated toxic intermediates (e.g., formaldehyde) for sensitive downstream enzymes. This could lead to a preliminary scaling, a critical requirement for industrial translation. The successful tenfold increase in substrate concentration to 300 mM methanol, yielding a 37% carbon conversion to L-valine and 45% to L-valine plus L-alanine, illustrates the cascade’s potential. Similarly, scaling the reaction volume 75-fold times from 200 µL to 15 mL for L-aspartic acid, while maintaining stable yields of 4.0 mM, underscores the adaptability of the modular design to larger systems. These achievements address a key challenge in biocatalysis: transitioning from proof-of-concept to industrial viability.

However, although this system shows immense potential, in this proof-of-concept, produced amino acid concentrations are still low. To get this system to work and produce the target amino acids from methanol, currently, very high enzyme concentrations, especially for the formaldehyde-fixing enzymes, had to be used due to their low activity and low specificity on formaldehyde (Supplementary Table 5). This step is crucial in the one-pot system as formaldehyde has strong inactivating effects on enzymes in already small concentrations^47^. Enzyme engineering is a valuable technique for stabilizing and enhancing enzyme activity. Formolase has been engineered to include improved variants, such as FLSM3^4^. Recently, a new formolase variant FLSM9, was released, which showed further improvement in activity and a 3.4-fold increase in *k*_*cat*_/*K*_m_^48^. Despite the significant interest in FLS in research involving rapid advancements, substantial progress is still necessary to achieve relevant performance for synthesizing industrially relevant titers.

While these enzymes will be improved through engineering campaigns in the future, one possibility to increase the titer of the product is to physically split the production of the C2 and C3 intermediate from the amino acid production. This circumvents the pathway enzymes are affected by toxic intermediates or uneven distribution of precursor molecules as a possible bottleneck in the methanol to L-glutamic acid/L-proline system. Indeed, several studies show that enriching dihydroxyacetone or glycolaldehyde in a physically separated step can be a viable option for production^25,49,50^. Similar to this, the two-pot system used in this study could benefit from the conversion of higher substrate titer to generate a ten-fold increase in the titer of L-valine compared to the one-pot cascade system. Still, the time frame, the conversion efficiency and the titer itself need further improvement. Here, besides enzyme engineering, prototyping guided by machine learning^51^ could aid in enhancing the ratios of enzymes, cofactors, and additives to discover the optimal conditions for producing the desired amino acid with a yield greater than 90%.

When the activity and stability of the enzymes can be brought to economically viable numbers (i.e., TTN of enzymes ca. 10^6^, activity in the range of 50 U mg ^-1^, production cost of enzymes at 1000 €/kg), the following scenario can be envisioned:

Using photovoltaics from 1 ha of more or less arable land, depending on the location on the planet, between 100 and 200 metric ton of methanol can be obtained, even including the energy necessary for CO_2_ air capture (Supplementary Table 10). The amino acid production has the potential for a high mass conversion of methanol (being > 90% for alanine and still above 54% for the example of valine). With this energy available, in a very rough estimate that does not include any process optimization or the in- and output of heat energy, up to 160 to/ha of alanine or 110 to/ha of valine could be produced, being a multiple of the amount of plant material, such as soy or wheat, that would be obtained under optimal conditions.

The resource-efficient supply of proteins and amino acids as one of the macronutrients is a key aspect in making humans dwelling on this planet more sustainable. The supply of protein is considered the greatest challenge for the sustainable nutrition of a growing world population^52^. Grazing animals have a huge carbon footprint due to deforestation as well as methane emissions, thus substantially contributing to global warming. Meat production today is dominated by feeding animals with plant proteins, such as soy. More sustainable and increasingly done is directly feeding them amino acids that are produced chemically or by fermentation^53,54^. In addition, cultured meat, as the most recent solution to animal-free protein, requires, among others, amino acids in the growth medium. Therefore, being able to also provide amino acids directly from carbon dioxide and renewable energy would allow a sustainable supply of all classes of macronutrients.

As demonstrated in this study, amino acid supply from carbon dioxide and electricity, and such a power-based protein/amino acid production as part of a methanol-based economy, has the potential to free more than 90% of the land area, making the human food production much more sustainable. With the advent of agrivoltaics, the first step towards this goal has been made.

## Methods

### Chemicals and reagents

All chemicals used were purchased from VWR, Carl-Roth, Serva, Roche, Sigma-Aldrich, Merck, Alfa-Aesar, and Fisher Scientific. KGSA was prepared as described by Sutiono et al.^55^. Alcohol oxidase (*pp*AOX) of *Pichia pastoris* was purchased from Sigma-Aldrich. Catalase (CAT) of *Corynebacterium glutamicum* was purchased from Sigma-Aldrich. Malate dehydrogenase (porcine heart) was purchased from Sigma-Aldrich. Glutamate dehydrogenase (bovine liver) was purchased from Sigma-Aldrich. Formate dehydrogenase (*Candida Boindinii*) was purchased from Megazymes. Horseradish peroxidase (HRP) was purchased from Sigma-Aldrich.

All gases, H_2_ (Linde, 5.0) and CO_2_ (Linde, 4.5) were used as purchased.

### Database search

For the analysis of pathways and suitable enzyme candidates for our systems, KEGG database^31,56,57^, BRENDA database^33^ and Biocyc database^32^ were used.

### Thermodynamic analysis via eQuilibrator software

For the thermodynamic analysis of the different reactions (Fig. 2a-i and Supplementary Table 2), the eQuilibrator 3.0 web tool^34^ was used with default conditions (pH: 7.5, pMg: 3.0, Ionic strength: 0.25).

### Bacterial strains

For cloning strain, *Escherichia coli* (*E. coli*) NEB® Turbo cells from New England Biolabs GmbH (NEB). were utilized. *E. coli* BL21 (DE3) (Novagen) was utilized for the expression of heterologous proteins. For the heterologous expression of *so*GLOX, *E. coli* Lemo21 (DE3) cells (NEB) were used.

### Gene manipulation

Plasmids pET28a- and pET24a (Novagen) were used as vectors. Phusion high fidelity polymerase (NEB) was used to amplify target genes with restriction sites from genomic DNA. Restriction ligation cloning or Golden Gate cloning using *Bsa*I was used for the integration of target genes into the plasmids. Newly designed and previously created constructs are listed in Supplementary Table 1. Primer sequences for new constructs are listed in Table 2. Synthetic gene sequences for altered genes used in this study are listed in Supplementary Table 11. GALS gene was purchased from GeneArt™, *glox* gene was purchased from Twist Bioscience. The *gapn* gene was purchased from Twist Bioscience. The bacterial strain *Bacillus subtilis* (DSM 402) was purchased from DSMZ German Collection of Microorganisms and Cell Cultures GmbH (Braunschweig, Germany), the bacterial strain *Escherichia coli* TOP10 was purchased from Invitrogen AG; and the bacterial strain *Geobacillus stearothermophilus* (ATCC 7953) was purchased from Merck for the extraction of genomic DNA (gDNA). The bacterial strain *Variovorax paradoxus* EPS was kindly provided by Prof. Dirk Tischler (Ruhr University Bochum). DNA sequences of all enzymes expressed, purified, and used in this work are provided in a separate Supporting Information file.

**Table 2:** Oligonucleotide primers used in this study with restriction sites highlighted

Gene	Forward primer	Reverse primer
*ecpepck*	GC**CATATG**CGCGTTAACAATGGTTTGACC	TA**AAGCTT**CAGTTTCGGACCAGCCGCTAC
*ecproc*	AAAAAA**GGTCTC**ACATGGAAAAGAAAATCGGTTTTATTGG	TTTTTT**GGTCTC**TCTTATCAGGATTTGCTGAGTTTTTCTG
*vpaspdh*	AACA**CATATG**ACCGCCATCACACGCATCGC	CCG**GAGCTC**TCAGATGGCGAGAGGCGCGAC
*bsleudh*	AT**GCTAGC**ATGGAACTTTTTAAATATATGGAG	CA**GAGCTC**TTAACGTCTGCTTAATACAC
*soglox*	GCAG**CATATG**GATGGAAATTACCAACGTG	GACGA**CTCGAG**TTACAGACGGGCAACTGCACGGCTG
*ecltae*	GACAG**CATATG**ATTGATTTACGCAGTGATACCG	GCGA**CTCGAG**TTAACGCGCCAGGAATGCACGC

### Protein production

*E. coli* BL21 (DE3) functions as a host strain for heterologous protein expression. Competent *E. coli* BL21 (DE3) were transformed with the appropriate plasmid harboring the gene of interest by a heat shock protocol. Cells were thawed on ice and the plasmid added incubated for 30 min and afterwards a heat shock at 42 °C was performed for 45 sec. Cells were immediately incubated on ice for 2 min. Afterwards 1 mL SOC medium was added and cells were incubated at 37 °C for 45 min. Cells were plated on LB-agar plates containing kanamycin. Expression of proteins was carried out in two steps. First 5–20 mL of LB-medium containing 100 µg mL^-1^ kanamycin was inoculated with a colony from a LB-agar plate and incubated overnight at 37 °C and 150–300 rpm. Main cultures were cultivated in 2 L or 5 L baffled shaking flasks containing either 400 mL or 1 L of 20% (v v^-1^) ZYP-5052 auto induction medium^58^ with 100 µg mL^-1^ kanamycin or terrific broth medium with 100 µg mL^-1^ kanamycin. The main culture was inoculated to an OD_600_ of 0.05 with the overnight culture and incubated at 37 °C and 120 or 90 rpm until an OD_600_ of 0.6–0.8. By using terrific broth medium, expression was induced by adding 0.5 mM of Isopropyl-ß-D-thiogalactopyranosid (IPTG). Temperature was reduced to 25 °C or 16 °C (Supplementary Table 1). After 20 h of cultivation, *E. coli* cells were centrifuged (4000 g, 30 min) and stored at –20 °C until purification.

### Glycolate oxidase production

For the heterologous expression of *so*GLOX, competent *E. coli* Lemo21 (DE3) cells were transformed with pET28a harboring the *glox* gene, similar to the procedure described before, and plated on LB-agar plates containing kanamycin and chloramphenicol. First 5 - 10 mL of LB-medium containing 50 µg mL^-1^ kanamycin and 35 µg mL^-1^ chloramphenicol was inoculated with a colony from a LB-agar plate and incubated overnight at 37 °C and 150 - 300 rpm. Main cultures were cultivated in 1 L or 2 L baffled shaking flasks containing either 200 mL or 400 mL terrific broth medium with 50 µg mL^-1^ kanamycin and 35 µg mL^-1^ chloramphenicol. The main culture was inoculated to an OD_600_ of 0.05 with the overnight culture and incubated at 37 °C and 120 rpm. After reaching an OD_600_ of 0.6–0.8, 0.1 mM IPTG was added to the culture and the temperature was reduced to 25 °C. Cells were harvested after 24 h of incubation and stored at –20 °C.

### High-volume production of FLSM3

For the upscaled L-aspartic acid cascade, FLSM3 was expressed in 2.5 L Ultra Yield flasks (Thomson). Main cultures containing 1 L terrific broth medium with 100 µg mL^-1^ kanamycin were inoculated with 100 mL pre-culture and incubated at 37 °C and 180 rpm until an OD_600_ of 4 – 9. Protein expression was induced by adding 1 mM of IPTG and cultures were incubated at 25 °C and 180 rpm. After 20 h of cultivation, *E. coli* cells were centrifuged (4000 g, 30 min) and stored at −20 °C until purification.

### Protein purification

Cell lysates were prepared on ice using ultrasonication (Ultraschallprozessor UIS250V Hielscher Ultrasonic GmbH). The cell pellets were resuspended in 50 mM HEPES pH 8.0, 500 mM NaCl, 20 mM imidazole in a 50 mL falcon tube. This way, a solution of a maximum of 20% (w v^-1^) of the cells was prepared. Lysis was conducted via sonication with an amplitude of 80% and a cycle length of 0.6-sec puls and 0.4-sec pause for two times 8 - 10 min and centrifuged afterward (4 °C, 40000 g, 30 min).

All proteins containing a His_6_ tag were purified using an ÄKTApurifier system (GE Healthcare) equipped with either a 1 mL or a 5 mL HisTrapFF column (Cytiva). The column was first equilibrated with binding buffer (50 mM HEPES pH 8.0, 20 mM imidazole, 500 mM NaCl). The supernatant was then applied. A wash step with binding buffer was used to remove unbound proteins. To elute the His_6_-tagged proteins from the column, the imidazole concentration was increased to 500 mM. The protein-containing eluate was collected. Buffer was exchanged to 50 mM HEPES pH 7.5 with either a HiPrep 26/10 or a PD10 desalting column (Cytiva). The purified protein solution was flash frozen in liquid nitrogen and stored at –80 °C.

In addition to the purification described above, similar to Nowak et al.^59^. the NADH oxidase was incubated with an excess of FAD for 15 minutes at 37 °C. Precipitated protein was removed by centrifugation, and unbound FAD was removed by a second desalting step with 50 mM HEPES pH 7.5.

For FLSM3 used in the upscaled L-aspartic acid cascade, lysis of 1.5 L resuspended cells from 300 g biomass was performed using a homogenizer (1.5 kbar, 8 °C) instead of an ultrasound treatment. IMAC of the cleared cell lysate was performed on a 20 mL HisPrep™ FF 16/10 column (VWR, Cytiva). The flow-through was reloaded for higher protein yields. The buffer of the pooled elution fractions was exchanged by three rounds of dialysis using 5 L 50 mM HEPES pH 7.5 buffer at 4 °C and 100 rpm over 20 h.

For *tk*GAPN used in upscaled L-aspartic acid cascade, an additional heat treatment was applied before centrifugation (65 °C, 45 min).

### Enzyme kinetic measurements

Enzyme kinetics (Supplementary Table 3) were determined in 200 µL reactions at 30 °C with 100 mM HEPES pH 7.5, if not stated otherwise. Additionally, further cofactors and additives were used according to the analyzed enzymes and coupling enzymes. Reactions were started by addition of the target substrate and measured by using a Biotek epoch-2 microplate spectrophotometer (Agilent, software Gen5 3.16).

#### Aldehyde dehydrogenase

Aldehyde dehydrogenase variant (*ta*ALDH variant M42) was assayed by following reduction of NAD^+^ in 200 µL. Reactions containing 5 mM NAD^+^, 0–20 mM glycolaldehyde and 0.05 mg mL^-1^ M42. Reactions were started by adding glycolaldehyde to the reaction mixture.

#### Glycolate oxidase

Glycolate oxidase (GLOX) was assayed via a coupled assay similar to Holt et al.^60^. In this assay, the oxidation of the substrate glycolic acid is coupled to the oxidation of 4-aminoantipyrine via horseradish peroxidase and a further reaction of the oxidized form of 4-aminoantipyrine with vanillic acid. Activity of glycolate oxidase was measured by following the absorbance increase at 500 nm. The reaction mixture contained 0.75 mM 4-AAP, 1 mM vanillic acid, 5 mM MgCl_2_, 0–10 mM glycolic acid, 2 U horseradish peroxidase (HRP) and 3.0 µg mL^-1^ *so*GLOX. The reaction was started by adding glycolic acid to the reaction mixture.

#### Alanine dehydrogenase

Alanine dehydrogenase (AlaDH) was assayed by directly measuring the oxidation of NADH. Standard reaction contained 200 mM (NH_4_)_2_SO_4_, 0.3 mM NADH, 50–0.00 mM glyoxylic acid and 0.46 µg mL^-1^ *bs*AlaDH. The reaction was started by addition of glyoxylic acid to the reaction mixture.

#### Phosphoenolpyruvate carboxykinase

Phosphoenolpyruvate carboxykinase (PEPCK) was assayed in a coupled system with malate dehydrogenase by following the oxidation of NADH at 340 nm. The final reaction mixture contained 200 mM (NH_4_)HCO_3_, 2.5 mM ADP, 0.3 mM NADH, 5 mM MgCl_2_, 0.1 mM MnSO_4_, 0 - 35 mM phosphoenolpyruvic acid (PEP), 0.5 µg mL^-1^ *ec*PEPCK and 4 µg malate dehydrogenase. The reaction was started by the addition of PEP to the reaction mixture.

#### Aspartate dehydrogenase

Aspartate dehydrogenase (AspDH) was assayed by directly measuring the oxidation of NADH. The final reaction mixture contained 200 mM (NH_4_)_2_SO_4_, 5 mM MgCl_2_, 0 – 25 mM oxaloacetic acid (OAA) and 2.0 µg mL^-1^ *vp*AspDH. The reaction was started by the addition of OAA to the reaction mixture.

#### Acetolactate synthase

Acetolactate synthase (ALS) was assayed in a coupled system with ketol-acid reductoisomerase (*gs*KARI) by following the oxidation of NADH at 340 nm. The final reaction mixture contained 5 mM MgCl_2_, 0.3 mM NADH, 0.5 mM thiamin pyrophosphate (TPP), 0–20 mM pyruvic acid, 0.7 mg mL^-1^ *gs*KARI and 2.1 µg mL^-1^ *bs*ALS. The reaction was started by the addition of pyruvic acid to the reaction mixture.

#### Ketol-acid reductoisomerase

Ketol-acid reductoisomerase (KARI) was assayed in a coupled system with acetolactate synthase (*bs*ALS) by following the oxidation of NADH at 340 nm. The final reaction mixture contained 5 mM MgCl_2_, 0.3 mM NADH, 0.5 mM TPP, 0 - 25 mM pyruvic acid, 80 µg mL^-1^ *bs*ALS and 38 µg mL^-1^ *gs*KARI. The reaction mixture containing all components except for *gs*KARI was incubated at 30 °C for 10 min. After the incubation, *gs*KARI was added and the absorption was monitored at 340 nm.

#### Dihydroxy-acid dehydratase

Dihydroxy-acid dehydratase (DHAD) was assayed in a coupled system with leucine dehydrogenase (*bs*LeuDH) by following the oxidation of NADH at 340 nm. The final reaction mixture contained 200 mM (NH_4_)_2_SO_4_, 5 mM MgCl_2_, 0.3 mM NADH, 0 - 20 mM 2,3-dihydroxyisovaleric acid, 24 µg mL^-1^ *bs*LeuDH and 1.3 µg mL^-1^ *st*DHAD. Reaction was started by adding 2,3-dihydroxyisovaleric acid to the reaction mixture.

#### Leucine dehydrogenase

Leucine dehydrogenase (LeuDH) was assayed by following the oxidation of NADH at 340 nm. The final reaction mixture contained 200 mM (NH_4_)_2_SO_4_, 5 mM MgCl_2_, 0.3 mM NADH, 0–20 mM pyruvic acid or 0–10 mM 2-ketoisovaleric acid or 0–200 mM α-ketoglutaric acid semialdehyde and 2.4 µg mL^-1^ *bs*LeuDH or 0.24 µg mL^-1^ *bs*LeuDH or 4.0 µg mL^-1^ *bs*LeuDH. Reaction was started by adding pyruvic acid or 2-ketoisovaleric acid or α-ketoglutaric acid semialdehyde to the reaction mixture.

#### 2-Dehydro-3-deoxy-D-gluconate aldolase

2-dehydro-3-deoxy-D-gluconate aldolase (KDGA) was assayed in a coupled system with 2-keto-3-deoxy-D-xylonate dehydratase (*pp*D-KDPD) and α-ketoglutaric acid semialdehyde dehydrogenase (*pp*KGSALDH) by following the reduction of NAD^+^. Due to the two substrates of *pt*KDGA, the reaction was conducted one time with a fixed glycolaldehyde and one time with a fixed pyruvic acid concentration. The final reaction mixture contained 2.5 mM NAD^+^, 10 mM MgCl_2_, 0.5 mM TPP, 0–50 mM glycolaldehyde (50 mM), 30 mM pyruvic acid (0–30 mM pyruvic acid), 0.1 mg mL^-1^ *pp*D-KDPD, 0.2 mg mL^-1^ *pp*KGSALDH and 1.7 µg mL^-1^ *pt*KDGA. The reaction was started by the addition of glycolaldehyde or pyruvic acid to the reaction mixture.

### Further enzyme activity measurements

#### Amino acid dehydrogenases

Amino acid dehydrogenases (*bs*AlaDH, *vp*AspDH, *bs*LeuDH) were assayed by following the oxidation of NADH in 200 µL. Reactions contained 0.3 mM NADH, 10 mM hydroxypyruvic acid (*bs*AlaDH), 10 mM 2-ketoisovaleric acid (*bs*AlaDH), 5 mM α-ketoglutaric acid semialdehyde (*vp*AspDH) or 5 mM 2-keto-4-hydroxybutyric acid (*bs*LeuDH) and 200 mM (NH_4_)_2_SO_4_. Reactions were started by adding the corresponding amino acid dehydrogenase to the reaction mixture.

### Amino acid cascade from gylcolaldehyde and dihydroxyacetone

#### Glycine and serine

The cascade setup for glycine was set as follows. In a total volume of 100 µL of reaction solution, 10 mM glycolaldehyde, 0.5 mM TPP, 200 mM (NH_4_)_2_SO_4_, 100 mM HEPES pH 7.5, 10 mM MgCl_2_, 5 mM NAD^+^ were added to a 2 mL reaction tube. After mixing of the compounds, the enzyme cocktail (Supplementary Table 6) was added and placed into a thermoshaker with 500 rpm at 30 °C. Samples were taken by adding 10 µL of the reaction solution to 90 µL of MilliQ water and heated for 10 min at 95 °C to precipitate the enzyme.

For the serine cascade to the above-described setup 10 mM formaldehyde was added to the reaction mixture before addition of the enzyme mix (Supplementary Table 6).

#### L-Aspartic acid

The cascade setup for L-aspartic acid was set as follows. In a total volume of 100 µL of reaction solution, 10 mM dihydroxyacetone, 0.5 mM TPP, 0.5 mM glucose 1-phosphate, 200 mM (NH_4_)HCO_3_ (400 mM (NH_4_)HCO_3_ for the optimized system), 100 mM HEPES pH 7.5, 0.1 mM MnSO_4_, 10 mM MgCl_2_, 2 mM ATP and 5 mM NAD^+^ were added to a 2 mL reaction tube. After mixing of the compounds, the enzyme cocktail (Supplementary Table 7) was added and placed into a thermoshaker with 500 rpm at 30 °C. Samples were taken by adding 10 µL of the reaction solution to 90 µL of MilliQ water and heated for 10 min at 95 °C to precipitate the enzyme.

#### L-Valine

The cascade setup for L-valine was set as follows. In a total volume of 100 µL of reaction solution, 10 mM dihydroxyacetone, 0.5 mM TPP, 0.5 mM glucose 1-phosphate, 200 mM (NH_4_)_2_SO_4_, 100 mM HEPES pH 7.5, 0.1 mM MnSO_4_, 10 mM MgCl_2_, 2 mM ATP and 5 mM NAD^+^ were added to a 2 mL reaction tube. After mixing of the compounds, the enzyme cocktail (Supplementary Table 7) was added and placed into a thermoshaker with 500 rpm at 30 °C. Samples were taken by adding 10 µL of the reaction solution to 90 µL of MilliQ water and heated for 10 min at 95 °C to precipitate the enzyme.

#### L-Glutamic acid and L-proline

The cascade setup for L-glutamic acid was set as follows. In a total volume of 100 µL of reaction solution, 10 mM dihydroxyacetone, 10 mM glycolaldehyde, 0.5 mM TPP, 0.5 mM glucose 1-phosphate, 200 mM (NH_4_)_2_SO_4_, 100 mM HEPES pH 7.5, 0.1 mM MnSO_4_, 10 mM MgCl_2_, 2 mM ATP and 5 mM NAD^+^ were added to a 2 mL reaction tube. After mixing of the compounds, the enzyme cocktail (Supplementary Table 7) was added and placed into a thermoshaker with 500 rpm at 30 °C. Samples were taken by adding 10 µL of the reaction solution to 90 µL of MilliQ water and heated for 10 min at 95 °C to precipitate the enzyme.

For the L-proline cascade to the above-described setup, 10 mM formic acid was added to the reaction mixture before addition of the enzyme mix.

### Methanol synthesis from CO_2_

Methanol was synthesized in a 4-channel plug flow reactor system equipped with online GC analytics. The core of the system are four stainless steel tubes with an inner diameter of 4 mm. Two of them were loaded with 21.5 and 194 mg (250 μm spheres) of an industrial CuZnAlO_x_ catalyst diluted 1:10 with SiC. Then, they were placed into a furnace, which can be heated isothermally up to 500 °C. Mass flow controllers (Bronkhorst) allow for adjusting the volume flows of CO_2_ (0-25 mL min^-1^) and H_2_ (0-80 mL min^-1^), which are mixed before entering the reactors. After the reactor, a back pressure regulator maintains a set pressure in the reactor. After that, an automatically driven switch valve allows a sample to be sent through a heated transfer line to a GC for analysis. Samples, which were not sent to the GC dropped into an open flask after cooling to room temperature and before venting the gases. Samples for analysis were kept in the gas phase by a heated transfer line and were sent to a Shimadzu GC-MS QP 2010 SE system with two analytical lines, one equipped with a CP-Molesieve 5 A (Agilent) and a PoraPlot Q (Agilent) column to analyze permanent gases via a TCD and a second equipped with an HP-1 (Agilent) column and an MS as detector. All relevant products were recorded: CO_2_, CO, and CH_3_OH.

### Amino acid cascades from methanol

#### Glycine and serine

Glycine and serine cascade reactions were set up in 200 µL scale in 2 mL tubes. 100 mM HEPES pH 7.5, 200 mM (NH_4_)_2_SO_4_, 5 mM NAD^+^, 10 mM MgCl_2_, 0.5 mM TPP and 30 mM methanol were mixed. Before the addition of enzymes to the reaction mixture, enzymes were concentrated using a previously washed (50 mM HEPES pH 7.5) centrifugal filter with modified PES 10 kDa (VWR). Reactions were started with addition of target enzyme composition and incubated at 30 °C, 500 rpm (Supplementary Table 6, Supplementary Table 8). Samples were taken by adding 30 µL of the reaction solution to 270 µL of Milli-Q water in a centrifugal filter with modified PES 10 kDa (VWR) and centrifuged (10 min, 10 °C, and 10000 g).

#### L-Aspartic acid

L-Aspartic acid cascade was set in a total volume of 200 µL scale in 2 mL tubes. 30 mM methanol, 0.5 mM TPP, 0.5 mM glucose 1-phosphate, 400 mM (NH_4_)HCO_3_, 100 mM HEPES pH 7.5, 0.1 mM MnSO_4_, 10 mM MgCl_2_, 2 mM ATP and 5 mM NAD^+^ were mixed. Before addition of enzymes to the reaction mixture, enzymes were concentrated using a previously washed (50 mM HEPES pH 7.5) centrifugal filter with modified PES 10 kDa (VWR). Reactions were started with the addition of target enzyme composition and incubated at 30 °C, 500 rpm (Supplementary Table 7, Supplementary Table 8). Samples were taken by adding 30 µL of the reaction solution to 270 µL of MilliQ water in a centrifugal filter with modified PES 10 kDa (VWR) and centrifuged (10 min, 10 °C, and 10,000 g).

#### L-Valine

L-Valine cascade was set in a total volume of 200 µL scale in 2 mL tubes. 30 mM methanol, 0.5 mM TPP, 0.5 mM glucose 1-phosphate, 200 mM (NH_4_)_2_SO_4_, 100 mM HEPES pH 7.5, 0.1 mM MnSO_4_, 10 mM MgCl_2_, 2 mM ATP and 5 mM NAD^+^ were mixed. Before the addition of enzymes to the reaction mixture, enzymes were concentrated using a previously washed (50 mM HEPES, pH 7.5) centrifugal filter with modified PES 10 kDa (VWR). Reactions were started with the addition of target enzyme composition and incubated at 30 °C, 500 rpm (Supplementary Table 7, Supplementary Table 8). Samples were taken by adding 30 µL of the reaction solution to 270 µL of MilliQ water in a centrifugal filter with modified PES 10 kDa (VWR) and centrifuged (10 min, 10 °C, and 10000 g).

#### L-Glutamic acid and L-proline

L-Glutamic acid and proline cascade was set in a total volume of 200 µL scale in 2 mL tubes. 30 mM methanol, 0.5 mM TPP, 0.5 mM glucose 1-phosphate, 200 (NH_4_)_2_SO_4_, 100 mM HEPES pH 7.5, 0.1 mM MnSO_4_, 10 mM MgCl_2_, 2 mM ATP and 5 mM NAD^+^ were mixed. Before addition of enzymes to the reaction mixture, enzymes were concentrated using previously washed (50 mM HEPES pH 7.5) centrifugal filter with modified PES 10 kDa (VWR). Reactions were started with addition of target enzyme composition and incubated at 30 °C, 500 rpm (Supplementary Table 7, Supplementary Table 8). Samples were taken by adding 30 µL of the reaction solution to 270 µL of MilliQ water in a centrifugal filter with modified PES 10 kDa (VWR) and centrifuged (10 min, 10 °C, and 10,000 g). For the Proline cascade to the above-described setup, 10 mM formic acid was added to the reaction mixture before addition of the enzyme mix.

### L-Valine production from CO_2_-derived methanol and ammonium

L-Valine cascade was set in a total volume of 1000 µL in 50 mL tubes. 300 mM CO_2_-based methanol, 0.5 mM TPP, 100 mM HEPES pH 7.5, 0.5 mM MnSO_4_, 10 mM MgCl_2_, were mixed before the addition of 5.3 mg mL ^-1^ FLSM3 and 1 µL catalase (*Corynebacterium glutamicum*) to the reaction mixture. Reactions were started with the addition of 0.1 mg mL ^-1^ alcohol oxidase (*Pichia pastoris*) and incubated at 30 °C on a nutating shaker (VWR) at 500 rpm. Samples were taken by adding 50 µL of the reaction solution to 200 µL or 250 µL of MilliQ water in a centrifugal filter with modified PES 10 kDa (VWR) and centrifuged (10 min, 10 °C, and 10,000 g). Samples were taken until 25 h. After 25 h of reaction time, 500 µL of the reaction solution was added to a freshly prepared master mix of the L-valine cascade enzymes, cofactors and additives. Final concentration of enzymes was similar to the one-pot system and is listed in Supplementary Table 7 (L-valine). Final concentrations of additives and cofactors were 0.5 mM TPP, 0.5 mM glucose 1-phosphate, 200 mM (NH_4_)_2_SO_4_, 100 mM HEPES pH 7.5, 0.5 mM MnSO_4_, 10 mM MgCl_2_, 2 mM ATP and 5 mM NAD^+^. Samples were taken by adding 100 µL of the reaction solution to 200 µL of MilliQ water in a centrifugal filter with modified PES 10 kDa (VWR) and centrifuged (10 min, 10 °C, and 10000 g). Control experiments were conducted similarly without the addition of alcohol oxidase.

### L-Aspartic acid production from CO_2_-derived methanol and ammonium

L-Aspartic acid cascade was set in a total volume of 15 mL in 500 mL sterile Erlenmeyer flask without chicanery, covered with aluminum foil. For the first part of the reaction with a total volume of 7 mL per replicate, 60 mM CO_2_-based methanol, 0.5 mM TPP, 100 mM HEPES pH 7.5, 0.1 mM MnSO_4_, and 10 mM MgCl_2_ were mixed in a 250 mL sterile Erlenmeyer flask without chicanery covered with an aluminum lid. 5.3 mg mL^-1^ FLSM3 and 2500 U/mL catalase (*Corynebacterium glutamicum*) were added to the reaction mixture. Reactions in triplicates (*n* = 3 samples) were started with the addition of 0.1 mg mL^-1^ alcohol oxidase (*Pichia pastoris*) and incubated at 30 °C on a shaker at 100 rpm. Samples were taken by adding 50 µL of the reaction solution to 200 µL of MilliQ water in a centrifugal filter with modified PES 10 kDa (VWR) and centrifuged (10 min, 10 °C, and 10,000 g). Samples were taken until 25 h. After 25 h of reaction time and pooling of the replicates of the first part of the reaction, 7.5 mL of the solution was added to a freshly prepared master mix of the L-aspartic acid cascade enzymes, cofactors and additives (one replicate). Final concentration of enzymes was similar to the one-pot system and are listed in Supplementary Table 7 (L-aspartic acid). Final concentrations of additives and cofactors were 0.5 mM TPP, 0.5 mM glucose 1-phosphate, 400 mM (NH_4_)HCO_3_ (added as powder), 100 mM HEPES pH 7.5, 0.1 mM MnSO_4_, 10 mM MgCl_2_, 2 mM ATP and 5 mM NAD^+^. Samples were taken by adding 50 µL of the reaction solution to 200 µL of MilliQ water in a centrifugal filter with modified PES 10 kDa (VWR) and centrifuged (10 min, 10 °C, and 10,000 g). The filtrate was stored at −20 °C until further analysis. For the first part of the reaction, the control experiment in triplicates (*n* = 3 samples) was conducted similarly without the addition of alcohol oxidase.

### Quantification of metabolites

#### Methanol quantification

Methanol was quantified using headspace gas chromatography with flame ionization detection (GC-FID) on a Thermo Scientific Trace GC Ultra instrument equipped with a Headspace Tri Plus autosampler. The Stabilwax column (30 m length, 0.25 mm internal diameter, 0.25 µm film thickness; Restek, Bellefonte, USA) was used with helium as the carrier gas, following the method of Willers et al. ^6^ based on Guterl et al.^61^. The oven temperature was held at 50 °C for 2 min and raised with 10 °C min^-1^ to 80 °C and held for 1 min. For the 30 mM methanol approach, 200 µL of the filtered cascade samples were added to a 10 mL headspace vial for analysis. Before injection, samples were incubated at 40 °C for 15 min. Injection was carried out via split mode with 10 mL min^-1^ flow, with an injection volume of 700 µL using headspace mode. The methanol standard curve (10–0.1 mM) was prepared in the cascade matrix and treated similarly to the cascade samples. For the 300 mM methanol approach, filtered samples were diluted 1:6 for the first part of the cascade (0–25 h) and 1:10 for the second part of the cascade (25 – 50 h) and treated similarly as described before. The methanol standard curve (15–0.5 mM) was prepared in the cascade matrix and treated similarly to the cascade samples. Quantification was carried out using the software Thermo Xcalibur 3.0.63.

#### Amino acid quantification

Amino acid quantification was carried out using an HPLC-system (Ultimate300 HPLC-system, Dionex Softron GmbH, Germaring, Germany) equipped with an AdvanceBio Amino Acid Analysis (AAA) column (3.0 ×100 mm, 2.7 µm), an AdvanceBio Amino Acid Analysis (AAA) guard column (4.6 ×5 mm, 2.7 µm) and an UltiMate 3000 RS fluorescence detector similar to the Agilent amino acid analysis protocol. Quantification was carried out using Chromeleon software (version 6.80, Thermo Fisher Scientific, Waltham, US). For the quantification of amino acids, samples containing primary amino acids (glycine, serine, L-aspartic acid, L-valine, L-glutamic acid) were derivatized with ortho-phthaladehyde (OPA) reagent and samples containing primary and secondary amino acids (L-proline) were derivatized with OPA reagent and 9-fluorenylmethyl chloroformate (Fmoc) solution previous to injection. For the online derivatization, one vial containing 0.1 M borate pH 10, one vial containing 1 M acetic acid, one vial containing freshly 1 to 10 diluted OPA reagent, and one vial containing freshly prepared Fmoc reagent, were placed into the autosampler, which was cooled to 10 °C. OPA reagent (Agilent) was diluted 1 to 10 in methanol. Fmoc solution (2.5 mg mL^-1^) was prepared in acetonitrile.

The online derivatization process followed this sequence: 1 µL of air was taken up, followed by 5 µL of 0.1 M borate pH 10, 1 µL of OPA, 1 µL of the sample, and 8 µL of air. This process was repeated 15 times, with 7 µL of air being released and taken up iteratively, and then 8 µL of air was released. 3 µL acetic acid (1 M) and 11 µL of air were taken up and 5 times 10 µL air released and taken up. Afterward, 11 µL of air were released and the sample was injected into the HPLC. The HPLC protocol was as follows. The column oven was set to 40 °C, the FLD flow cell temperature was set to 40 °C, the data collection rate was set to 10 Hz, wavelength set to λ_ex_: 337.0 nm and λ_em_: 442.0 nm and the flow rate set to 0.62 mL min^-1^. Starting conditions were 98% buffer A (10 mM Na_2_HPO_4_ and 10 mM Na_2_B_4_O_7_ pH 8.2) and 2% buffer B (45% methanol, 45% ACN, 10% Milli-Q water). Isocratic elution was conducted for 0.35 min. Afterwards, buffer B was raised from 2% to 57% in 13.05 min in a gradient. Buffer B was further raised to 100% in 0.1 min and run isocratically for 2.20 min. Starting conditions were restored by reducing buffer B to 2% in 0.1 min and run isocratic with 98% buffer A and 2% buffer B for 2.20 min. Overall, the method ran over a time period of 18 min.

The method was modified for the quantification of L-proline. After the first 15 mixing cycles, 1 µL Fmoc reagent and 8 µL air were taken up and mixed 15 times again as described above. After releasing 8 µL of air, 3 µL of acetic acid and 11 µL of air were added and the procedure continued as before. The HPLC method remained similar, but as a difference, the wavelength of the FLD was switched (λ_ex_: 260.0 nm, λ_em_: 325.0 nm) after 10.0 min of run time. The method was continued as described above. The isocratic run with 98% buffer A and 2% buffer B after 15.8 min was extended from 2.20 to 4.20 min. The total run time for the L-proline detection method was 20 min.

Samples were prepared as follows. 10–100 µL of 1:10 diluted sample was mixed with 10 µL of 1 mM L-tyrosine (in 0.1 M HCl) and adjusted to 1000 µL with MilliQ water. Amino acid standards were diluted in the cascade matrix to mimic matrix effects. The final concentrations of the amino acid standards were adjusted to 0.5 µM to 10 µM by preparing the standards similar to the samples, adding 10 µL of amino acid solution, 10 µL 1 mM L-tyrosine (in 0.1 M HCl), and adjusting the volume to 1000 µL with MilliQ water. Quantification of the 300 mM methanol approach was slightly adjusted, for a 96-well setup. Filtered samples were diluted 1:1000 or 1:1200 and treated similarly as described before.

#### Dihydroxyacetone quantification

Dihydroxyacetone of the L-valine cascade was quantified using HPLC^62^. The analysis was performed with an Ultimate-3000 HPLC system equipped with an autosampler and a diode array detector (Dionex Softron, Germering, Germany), using an ion exclusion column (Rezex ROA-Organic Acid H^+^(8%), Phenomenex, Aschaffenburg, Germany). Samples were diluted 1:2 (0–25 h) or 1:4 (25.1–50 h) in 2.5 mM H_2_SO_4_ (c_end_). The HPLC measurements were performed with isocratic elution with 2.5 mM H_2_SO_4_ at 70 °C for 30 min. Quantification was carried out using Chromeleon software (version 6.80, Thermo Fisher Scientific, Waltham, US). The concentration of dihydroxyacetone was calculated based on the area of the signal. By analysis of the second phase of L-valine production (after addition of the second enzyme, 25.1 - 50 hours), a raised dihydroxyacetone content in the 25.1 h sample compared to the 25 h sample (first step) was detected. However, no increase in methanol content (detected via GC-FID) or amino acid content (detected via HPLC fluorescence) was observed, which would have indicated inappropriate dilution of the cascade. However, a small peak was detected in the vicinity of dihydroxyacetone in the 37 h and 50 h samples. This signal influenced the dihydroxyacetone peak, resulting in the increased dihydroxyacetone concentration detected in the 25.1 h sample. The unknown signal was subtracted from the dihydroxyacetone signal in the samples (25.1–50 hours) to obtain the final values plotted in Fig. 3b.

In the C1-fixation module of the 15 mL L-aspartic acid cascade, dihydroxyacetone was quantified using a novel HPLC methodology relying on derivatization of carbonyls using 3-nitrophenylhydrazine. This methodology has recently been developed at the Chair of Chemistry of Biogenic Resources, Technical University Munich. Herein, we are referring to a future publication that will be published soon. Quantification was carried out using Chromeleon software 7.2.10 ES (Thermo).

### Metabolic profiling via HILIC LC-MS

Metabolic profiling via HILIC LC-MS was performed according to published methodologies^63,64^. Samples for the LC/Q-TOF-MS were prepared as follows^65,66^: 40 µL cascade sample was mixed with 2 µL 1 M ammonium acetate (pH 9.2), 8 µL 1 mM α-aminobutyric acid (internal standard) and 30 µL MilliQ H_2_O by vortexing shortly. After addition of 120 µL acetonitrile, samples were vortexed and chilled on ice for 10 min. Following centrifugation (10 min, 20,000 g, 4 °C), 150 µL supernatant were transferred to glass vials for measurement.

A bio-inert 1290 series UHPLC system interfaced to a Q-TOF mass spectrometer (G6546AA, Agilent Technologies) with a dual Agilent jet stream electrospray ion source was used for LC-MS analyses. Data acquisition was performed with MassHunter LC/MS Data Acquisition (version 10.1) from Agilent Technologies. Data evaluation was performed with MassHunter ProFinder (version 10.0) from Agilent Technologies using “batch targeted feature extraction” (Supplementary Table 12) relying on a personal target list of compounds including the substrate, intermediates and products of the enzyme cascades (Supplementary Table 13).

The mass spectrometer was operated in the low mass range (*m/z* 1700) applying the following source parameters: Gas temperature of 325 °C, drying gas flow rate of 8 L min^−1^, nebulizer pressure of 40 psi, sheath gas temperature of 350 °C, sheath gas flow rate of 12 L min^−1^, capillary voltage of 4000 V, nozzle voltage of 2000 V, and fragmentor voltage of 100 V. In line with the manufacturer´s recommendations, the instrument was auto tuned and calibrated with ESI-L tuning mix (Agilent Technologies). Online reference mass correction was ensured by continuous introduction of a solution of hexakis(1H, 1H, 3H-tetrafluoropropoxy)phosphazine (2.5 µM) and purine (4 µM) in acetonitrile/water, 95/5, v/v through the second sprayer of the dual ion source at a flow rate of 10 µL min^-1^ using an isocratic HPLC pump (G7110B, Agilent Technologies). Mass spectra were recorded in centroid mode with an acquisition rate of 1.4 spectra per second.

Cascade time point samples (5 µL injection volume) were separated on a SeQuant® ZIC®-pHILIC column (150 × 2.1 mm, 5 µm, Merck Millipore, Darmstadt, Germany) with a guard column (SeQuant® ZIC®-pHILIC, 20 × 2.1 mm, 5 µm, Merck Millipore, Darmstadt, Germany). As eluent A, 10 mM CH_3_COONH_4_ in MilliQ H_2_O (pH 9.2) with 90%, v/v acetonitrile and as eluent B, 10 mM CH_3_COONH_4_ in MilliQ H_2_O (pH 9.2) with 10%, v/v acetonitrile were used. The following binary gradient program was applied at a constant flow rate of 200 µL min^−1^: 0–1 min isocratic hold with 0% B, 1–31 min linear gradient from 0 to 75% B, 31 - 35 min linear gradient from 75 to 100% B, 35–40 min isocratic hold with 100% B, 40–50 min linear gradient from 100 to 0% B, 50–65 min isocratic hold with 0% B. The column temperature and autosampler temperature were maintained at 40 °C and 6 °C, respectively. Eluting compounds were monitored in negative ion mode over a *m/z* range of 25 - 1700.

Identity of the target compounds was confirmed with authentic standards. For absolute metabolite quantification, standard mixes in defined concentrations in HEPES reaction buffer were used for external calibration. Moreover, a purchased amino acid standard (Sigma Aldrich, AAS18) diluted to 100 mM in MilliQ water was measured along with each sequence as a quality control and system suitability standard. The result of its measurement was documented to track the performance of the machine and method. Lastly, a process blank, i.e., HEPES reaction buffer treated exactly like the experimental samples, was included in each sequence.

### Statistics & reproducibility

No statistical method was used to predetermine sample size. No data were excluded from the analyses, unless specified differently in the respective figure legends (i.e., when negligible LC signals were set to 0 to avoid showing negative concentrations and when only two out of three replicates were considered due to an outlier). The experiments were not randomized. The Investigators were not blinded to allocation during experiments and outcome assessment. Statistical analyses of the data were conducted by determining the mean values with error bars indicating the standard deviation of triplicates (*n* = 3 samples), unless specified differently. No statistical tests have been used.

### Reporting summary

Further information on research design is available in the Nature Portfolio Reporting Summary linked to this article.

## Supplementary information

Supplementary Information

Description of Additional Supplementary Files

Supplementary Data 1

Supplementary Data 2

Reporting Summary

Transparent Peer Review file

## Source data

Source Data

## Data Availability

All data are available in the main text or the supplementary information. Source data are provided with this paper.
