# Supplementary material for: Plug-and-play – enzymatic amino acid production from methanol and carbon dioxide
Source: Nat Commun. 2026 Jun 17;17:5363. doi: 10.1038/s41467-026-74522-x (PMC13276030; doi:10.1038/s41467-026-74522-x)
Supplement: Supplementary file 1 — Supplementary Information [file 41467_2026_74522_MOESM1_ESM.pdf]

Supplementary Information (SI) for

Plug and Play – Enzymatic Amino Acid Production from Methanol and Carbon Dioxide

Author list: Vivian Pascal Willers[1,*], Viktoria Lehmann[1,*], Susanne Müller[2], Ferdinand Vogelgsang[3], Arne Roth[3], Bastian Blombach[2,4], André Pick[5], and Volker Sieber[1,3,4,6,**]

Affiliations:

[1] Chair of Chemistry of Biogenic Resources, Campus Straubing for Biotechnology and Sustainability, Technical University Munich, 94315 Straubing, Germany.

[2] Professorship Microbial Biotechnology, Campus Straubing for Biotechnology and Sustainability, Technical University Munich, 94315 Straubing, Germany.

[3] Fraunhofer Institute for Interfacial Engineering and Biotechnology IGB, 94315 Straubing, Germany.

[4] SynBiofoundry@TUM, Technical University Munich, 94315 Straubing, Germany.

[5] CASCAT GmbH, Europaring 4, 94315 Straubing, Germany.

[6] School of Chemistry and Molecular Biosciences, The University of Queensland, St. Lucia 4072, Australia.

* These authors contributed equally.

** Corresponding author. Correspondence and requests for materials should be addressed to V.S. (email: sieber@tum.de).

Supplementary Note 1: Amino acid pathway construction and optimization

To build this Plug and Play enzyme system, the C2- and C3 intermediates glycolaldehyde and dihydroxyacetone have to be converted in the second module to yield pyruvic acid, phosphoenolpyruvic acid or glyoxylic acid and NADH. These molecules are then needed in the third module for the production of the corresponding amino acids by using different promiscuous NADH-dependent amino acid dehydrogenases. To do so, the general structure of this module is similar in all designed amino acid pathways having one NAD^+ dependent oxidation step surrounded by reactions leading to and away from this step towards the desired amino acid precursor molecule. By having dihydroxyacetone as intermediate, a possibility to generate pyruvic acid and NADH is the usage of the semi-phosphorylated ED-pathway. The semi phosphorylated ED-pathway opens up the way for pyruvic acid production in six enzymatic steps with an intrinsic ATP regeneration system. Dihydroxyacetone is phosphorylated by dihydroxyacetone kinase (DHAK). Dihydroxyacetone phosphate is further isomerized by triose phosphate isomerase (TPI) to glyceraldehyde 3-phosphate, which is oxidized by a non-phosphorylating glyceraldehyde 3-phosphate dehydrogenase (GAPN) *via* NAD^+ to 3-phosphoglyceric acid, which is then isomerized to 2-phosphoglyceric acid by phosphoglycerate mutase (PGM), converted to phosphoenolpyruvic acid by enolase (ENO) and finally converted to pyruvic acid by pyruvate kinase (PYK). However, by maintaining the ATP balance alternatively to pyruvic acid as target molecule in module 2, also oxaloacetic acid could be produced as direct precursor of the amino acid L-aspartic acid. In this variation of module 2, PYK is exchange by a phosphoenolpyruvic acid carboxy kinase (PEPCK), which restores ATP balance and leads the intermediate phosphoenolpyruvic acid directly to oxaloacetic acid.

Similarly, glycolaldehyde conversion is conducted, however, in this version of module 2, no ATP regeneration is needed as glycolaldehyde can be converted to the glycine precursor glyoxylic acid by having one NAD^+ dependent oxidation and one oxygen dependent oxidation.

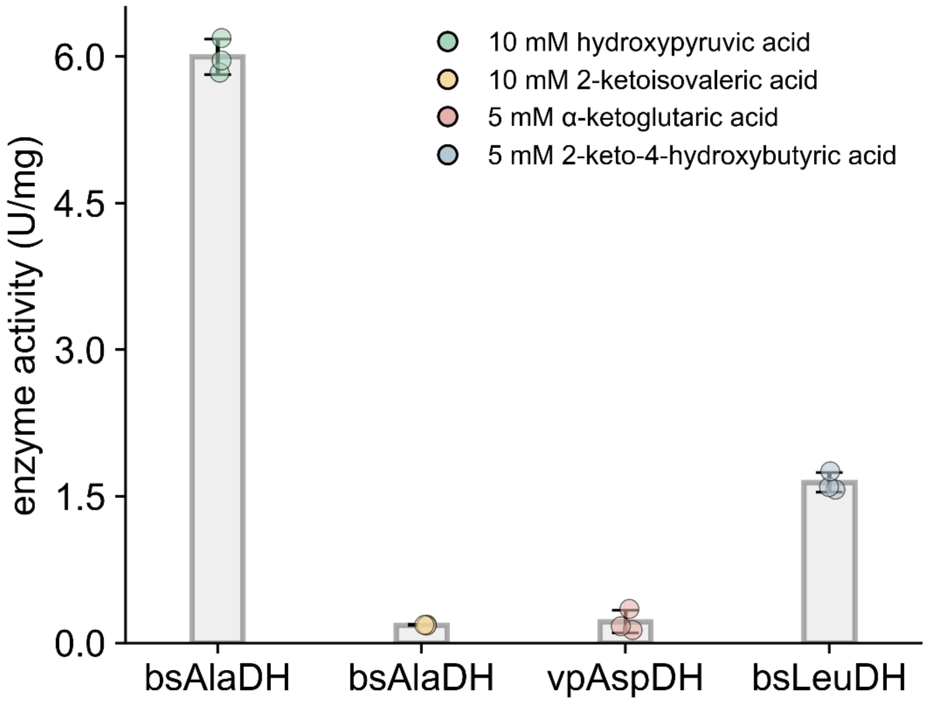

Supplementary Figure 1. Enzyme activities of amino acid dehydrogenases against further substrates. Data are presented as mean values with error bars indicating the standard deviation of triplicates (n = 3 samples). Individual data points are shown using overlaid dot plots. Source data for this figure are provided as a Source Data file.

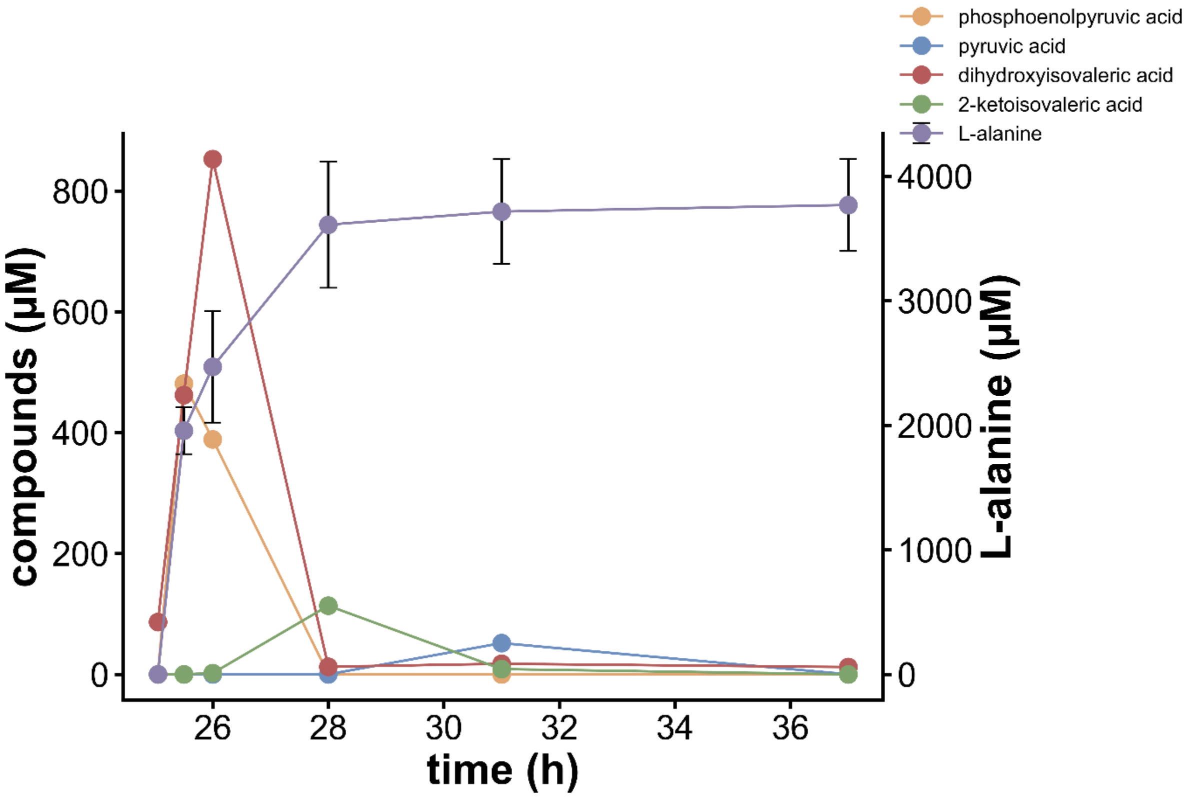

Supplementary Figure 2. Time curve of various metabolites (phosphoenolpyruvic acid, pyruvic acid, 2,3-dihydroxyisovaleric acid, 2-ketoisovaleric acid) measured *via* HILIC LC-MS and L-alanine as side product measured *via* HPLC of the scaled-up L-valine cascade. All other intermediates of the valine cascade including the remaining phosphorylated C3 bodies, acetolactic acid and its decarboxylated version, acetoin, were not found to be accumulated by HILIC LC-MS. HILIC LC-MS measurements of L-alanine and L-valine were consistent with quantification of both these compounds by HPLC. Quantification of phosphoenolpyruvic acid at 25.5 h and 26 h revealed clear accumulation, although the calculated and shown data points are slightly outside the calibration curve and should thus only be seen as trends. At the four remaining time points, phosphoenolpyruvic acid signal was either clearly below the calibration curve (25.25 h; thus, data point excluded) or negligible and was hence set to 0 µM (28 h, 31 h, 37 h; i.e., below the y-axis intercept, would have resulted in negative concentration values). Similarly, at time points other than 31 h, signals for pyruvic acid were negligible and were thus set to 0 µM. At 25 h, 25.5 h, and 37 h, signals for 2-ketoisovaleric acid were negligible and were thus set to 0 µM. For L-alanine, the signal at 25 h was negligible and was hence also set to 0 µM. Metabolomics *via* LC-MS was conducted as single measurement (n = 1 sample). Data for L-alanine production are presented as mean values with error bars indicating the standard deviation of triplicates (n = 3 samples). Source data for this figure are provided as a Source Data file.

Supplementary Table 1. Enzyme list. All enzymes used in this study are listed including their abbreviation, NCBI accession code, the organism they are derived from, and the conditions under which the enzymes were heterologously expressed in *E. coli*.

Enzyme	Abb.	NCBI Accession code	Organism	Construct	Expression conditions
**Alcohol oxidase**	AOX	XP_002494271.1	*Pichia pastoris*		
**Catalase**	CAT	BAV22054.1	*Corynebacterium glutamicum*		
**Formolase**	FLS		Computational designed protein	FLS-pET24a [S1]	
**Formolase-M3**	FLSM3		Computational designed protein	FLSM3-pET24a [S2]	TB-media 25 °C
**Glycolaldehyde synthase**	GALS		Computational designed protein	GALS-pET28a [S3]	ZY-media 25 °C
**Dihydroxyacetone kinase**	DHAK	WP_029604097.1	*Kozakia baliensis*	*kb*DHAK-pET28a [S2]	ZY-media 25 °C
**Triosephosphate isomerse**	TPI	WP_033015090.1	*Geobacillus stearothermophilus*	*gs*TPI-pET28a [S2]	ZY-media 25 °C
**Glyceraldehyde-3-phosphate dehydrogenase**	GAPN	BAD84894.1	*Thermococcus kodakarensis*	*tk*GAPN-pET28a [S2]	ZY-media 25 °C
**Aldehyde dehydrogenase**	ALDH (M42)	WP_010901221.1	*Thermoplasma acidophilum*	(M42)*ta*ALDH-pET28a [S4]	ZY-media 25 °C
**Glycolate oxidase**	GLOX	P05414	*Spinacia oleracea*	*so*GLOX-pET28a	TB-media 25 °C
**Phosphoglycerate mutase**	PGM	WP_033015095.1	*Geobacillus stearothermophilus*	*gs*PGM-pET28a [S2]	ZY-media 25 °C
**Enolase**	ENO	WP_004080719.1	*Thermotoga napthophila*	*tm*ENO-pET28a [S2]	ZY-media 25 °C
**Phosphoenolpyruvate carboxykinase**	PEPCK	A8A5L1.1	*Escherichia coli*	*ec*PEPCK-pET28a	ZY-media 25 °C
**Pyruvate kinase**	PYK	WP_010901306.1	*Thermoplasma acidophilum*	*ta*PYK-pET28a [S2]	ZY-media 25 °C
**Acetolactate synthase**	ALS	Q04789	*Bacillus subtilis*	*bs*ALS-pET28a [S5]	ZY-media 25 °C
**Ketol-acid reductoisomerase**	KARI	Q8RL86	*Geobacillus stearothermophilus*	*gs*KARI-pET28a [S6]	ZY-media 25 °C
**Dihydroxy acid dehydratase**	DHAD	WP_104358646.1	*Schlegelella thermodepolymerans*	*st*DHAD-pET28a [S7]	ZY-media 25 °C
**2-Dehydro-3-deoxy-D-gluconate aldolase**	KDGA	WP_048059513.1	*Picrophilus torridus*	pET28a-*pt*KDGA [S8]	ZY-media 25 °C
**2-Keto-3-deoxy-D-xylonate dehydratase**	D-KDPD	WP_010953745.1	*Pseudomonas putida*	*pp*D-KDPD-pET28a [S9]	ZY-media 25 °C
**2-Keto-3-deoxy-L-arabinonate dehydratase**	L-KDPD	WP_011616492.1	*Cupriavidus necator*	*cn*L-KDPD-pET28a [S9]	ZY-media 25 °C
**α-Ketoglutarate semialdehyde dehydrogenase**	KGSALDH	AAN66880.1	*Pseudomonas putida*	*pp*KGSALDH-pET28a [S10]	ZY-media 25 °C
**Pyrroline-5-carboxylate reductase**	PROC	P0A9L8.1	*Escherichia coli*	*ec*PROC-pET28a	ZY-media 25 °C
**Alanine dehydrogenase**	AlaDH	NP_391071.1	*Bacillus subtilis*	*bs*AlaDH-pET28a [S2]	ZY-media 16 °C
**Aspartate dehydrogenase**	AspDH	ADU37773.1	*Variovorax Paradoxus EPS*	*vp*AspDH-pET28a	ZY-media 25 °C
**Leucine dehydrogenase**	LeuDH	P54531.1	*Bacillus subtilis*	*bs*LeuDH-pET28a	ZY-media 25 °C
**L-Glutamate dehydrogenase**	GluDH	P00366	*Bos taurus*		
**Low specificity L-threonine aldolase**	LTA	P75823	*Escherichia coli*	*ec*LTA-pET28a	ZY-media 25 °C
**NADH oxidase**	NOX	CCB83530.1	*Lactobacillus pentosus*	*lp*NOX-pET28a [S11]	ZY-media 16 °C
**Formate dehydrogenase**	FDH	O13437.1	*Candida boidinii*		

*The NCBI reference sequences/PDB numbers presented in Supplementary Table 2 correspond to the wild type sequences.

Supplementary Table 2. Estimation of reaction Gibbs energy for 1 mM as standard concentrations of enzymatic amino acid system by using eQuilibrator 3.0 web tool in default mode with reaction steps and reaction input in eQuilibrator 3.0 web tool [S12].

Pathway	Reaction step	Reaction	Estimated $\Delta_rG'^m$ [kJ mol^{-1}]
**C1 fixation module**	1	Methanol(aq) + O_2(aq) ⇌ Formaldeyhde(aq) + H_2O_2(aq)	-98.9
	2a	2 Formaldeyhde(aq) ⇌ Glycolaldehyde(aq)	-28.7
	2b	3 Formaldeyhde(aq) ⇌ Dihydroxyacetone(aq)	-46.4
**Glycine**	1	Glycolaldehyde(aq) + NAD(aq) + H_2O(l) ⇌ Glycolic acid(aq) + NADH(aq)	-66.8
	2	Glycolic acid(aq) + O_2(aq) ⇌ Glyoxylic acid(aq) + H_2O_2(aq)	-88.3
	3	Glyoxylic acid(aq) + NADH(aq) + NH_4^+(aq) ⇌ NAD(aq) + H_2O(l) + Glycine(aq)	-24.8
**Serine**	1	Glycolaldehyde(aq) + NAD(aq) + H_2O(l) ⇌ Glycolic acid(aq) + NADH(aq)	-66.8
	2	Glycolic acid(aq) + O_2(aq) ⇌ Glyoxylic acid(aq) + H_2O_2(aq)	-88.3
	3	Glyoxylic acid(aq) + NADH(aq) + NH_4^+(aq) ⇌ NAD(aq) + H_2O(l) + Glycine(aq)	-24.8
	4	Glycine(aq) + Formaldehyde(aq) ⇌ Serine(aq)	-1.5
**L-Aspartic acid**	1	Dihydroxyacetone(aq) + ATP(aq) ⇌ Dihydroxyacetone phosphate(aq) + ADP(aq)	-14.9
	2	Dihydroxyacetone phosphate(aq) ⇌ D-Glyceraldehyde 3-phosphate(aq)	5.6
	3	D-Glyceraldehyde 3-phosphate(aq) + NAD(aq) + H_2O(l) ⇌ NADH(aq) + 3-Phosphoglyceric acid(aq)	-48.0
	4	3-Phosphoglyceric acid(aq) ⇌ 2-Phosphoglyceric acid(aq)	4.5
	5	2-Phosphoglyceric acid(aq) ⇌ Phosphoenolpyruvic acid(aq) + H_2O(l)	-3.8
	6	Phosphoenolpyruvic acid(aq) + ADP(aq) + CO_2(aq) ⇌ Oxaloacetic acid(aq) + ATP(aq)	6.5
	7	Oxaloacetic acid(aq) + NH_4^+(aq) + NADH(aq) ⇌ L-Aspartic acid(aq) + H_2O(l) + NAD(aq)	-19.1
**L-Valine**	1	Dihydroxyacetone(aq) + ATP(aq) ⇌ Dihydroxyacetone phosphate(aq) + ADP(aq)	-14.9
	2	Dihydroxyacetone phosphate(aq) ⇌ D-Glyceraldehyde 3-phosphate(aq)	5.6
	3	D-Glyceraldehyde 3-phosphate(aq) + NAD(aq) + H_2O(l) ⇌ NADH(aq) + 3-Phosphoglyceric acid(aq)	-48.0
	4	3-Phosphoglyceric acid(aq) ⇌ 2-Phosphoglyceric acid(aq)	4.5
	5	2-Phosphoglyceric acid(aq) ⇌ Phosphoenolpyruvic acid(aq) + H_2O(l)	-3.8
	6	Phosphoenolpyruvic acid(aq) + ADP(aq) ⇌ Pyruvic acid(aq) + ATP(aq)	-25.0
	7	2 Pyruvic acid(aq) ⇌ 2-Acetolactic acid(aq) + CO_2(aq)	-27.0
	8	2-Acetolactic acid(aq) + NADH(aq) ⇌ (2R)-2,3-Dihydroxyisovaleric acid(aq) + NAD(aq)	-15.6
	9	(2R)-2,3-Dihydroxyisovaleric acid(aq) ⇌ H_2O(l) + 2-Ketoisovaleric acid(aq)	-36.9
	10	2-Ketoisovaleric acid(aq) + NADH(aq) + NH_4^+(aq) ⇌ H_2O(l) + L-Valine(aq) + NAD(aq)	-18.0
**L-Glutamic acid**	1	Dihydroxyacetone(aq) + ATP(aq) ⇌ Dihydroxyacetone phosphate(aq) + ADP(aq)	-14.9
	2	Dihydroxyacetone phosphate(aq) ⇌ D-Glyceraldehyde 3-phosphate(aq)	5.6
	3	D-Glyceraldehyde 3-phosphate(aq) + NAD(aq) + H_2O(l) ⇌ NADH(aq) + 3-Phosphoglyceric acid(aq)	-48.0
	4	3-Phosphoglyceric acid(aq) ⇌ 2-Phosphoglyceric acid(aq)	4.5
	5	2-Phosphoglyceric acid(aq) ⇌ Phosphoenolpyruvic acid(aq) + H_2O(l)	-3.8
	6	Phosphoenolpyruvic acid(aq) + ADP(aq) ⇌ Pyruvic acid(aq) + ATP(aq)	-25.0
	7	Pyruvic acid(aq) + Glycolaldehyde(aq) ⇌ 2-Dehydro-3-deoxy-D-xylonic acid(aq)	2.5
	8	2-Dehydro-3-deoxy-D-xylonic acid(aq) ⇌ α-Ketoglutaric acid semialdehyde(aq) + H_2O(l)	-39.0
	9	α-Ketoglutaric acid semialdehyde(aq) + NAD(aq) + H_2O(l) ⇌ α-Ketoglutaric acid(aq) + NADH(aq)	-43.1
	10	α-Ketoglutaric acid(aq) + NADH(aq) + NH_4^+(aq) ⇌ NAD(aq) + H_2O(l) + L-Glutamic acid(aq)	-16.3
**L-Proline**	1	Dihydroxyacetone(aq) + ATP(aq) ⇌ Dihydroxyacetone phosphate(aq) + ADP(aq)	-14.9
	2	Dihydroxyacetone phosphate(aq) ⇌ D-Glyceraldehyde 3-phosphate(aq)	5.6
	3	D-Glyceraldehyde 3-phosphate(aq) + NAD(aq) + H_2O(l) ⇌ NADH(aq) + 3-Phosphoglyceric acid(aq)	-48.0
	4	3-Phosphoglyceric acid(aq) ⇌ 2-Phosphoglyceric acid(aq)	4.5
	5	2-Phosphoglyceric acid(aq) ⇌ Phosphoenolpyruvic acid(aq) + H_2O(l)	-3.8
	6	Phosphoenolpyruvic acid(aq) + ADP(aq) ⇌ Pyruvic acid(aq) + ATP(aq)	-25.0
	7	Pyruvic acid(aq) + Glycolaldehyde(aq) ⇌ 2-Dehydro-3-deoxy-D-xylonic acid(aq)	2.5
	8	2-Dehydro-3-deoxy-D-xylonic acid(aq) ⇌ α-Ketoglutaric acid semialdehyde(aq) + H_2O(l)	-39.0
	9	α-Ketoglutaric acid semialdehyde(aq) + NH_4^+(aq) + NADH(aq) ⇌ Glutamic acid 5-semialdehyde(aq) + NAD(aq) + H_2O(l)	-8.5

	10	Glutamic acid 5-semialdehyde(aq) ⇌ (S)-1-Pyrroline-5-carboxylic acid(aq) + H_2O(l)	-7.4
	11	NADH(aq) + (S)-1-Pyrroline-5-carboxylic acid(aq) ⇌ NAD (aq) + L-Proline(aq)	-44.5

Supplementary Table 3. Enzyme kinetics and enzyme activities measured in this study. Data are presented as mean values with errors representing the standard deviation of triplicates (n = 3 samples). Source data for this table are provided as a Source Data file.

Enzyme	v_{max} [U mg^{-1}]	K_m [mM]	Substrate	Fit
***ta*ALDH (M42)**	0.4 ± 0.0	6.9 ± 0.3	Glycolaldehyde	Michaelis Menten
ta*ALDH (M42)	v_{obs}: 1.4 x 10^{-3} ± 0.0	-	Formaldehyde	-
***so*GLOX**	4.9 ± 0.0	0.2 ± 0.0	Glycolic acid	Hill
***bs*AlaDH**	80.1 ± 2.2	17.6 ± 1.1	Glyoxylic acid	Michaelis Menten
***ec*PEPCK**	56.6 ± 8.4	4.7 ± 1.3	Phosphoenolpyruvic acid	Michaelis Menten
***vp*AspDH**	134.9 ± 12.0	6.5 ± 0.8	Oxaloacetic acid	Substrate inhibition (K_I: 44.8 ± 11.2)
***bs*ALS**	8.3 ± 0.2	0.5 ± 0.1	Pyruvic acid	Michaelis Menten
***gs*KARI**	0.4 ± 0.1	1.7 ± 0.1	Acetolactic acid**	Michaelis Menten
***st*DHAD**	0.8 ± 0.0	0.6 ± 0.0	2,3-Dihydroxyisovaleric acid	Michaelis Menten
***bs*LeuDH**	77.1 ± 2.5	0.4 ± 0.0	2-Ketoisovaleric acid	Substrate inhibition (K_I: 30.3 ± 5.1)
***bs*LeuDH**	2.3 ± 0.2	11.3 ± 2.3	Pyruvic acid	Michaelis Menten
***pt*KDGA**	2.4 ± 0.1	0.2 ± 0.0	Pyruvic acid	Michaelis Menten
***pt*KDGA**	2.6 ± 0.0	5.5 ± 0.1	Glycolaldehyde	Michaelis Menten
***bs*LeuDH**	11.2 ± 1.2	117.1 ± 21.6	α-Ketoglutaric acid semialdehyde	Michaelis Menten

*Activity (v_{obs}) on 10 mM formaldehyde of *ta*ALDH (M42).
**Due to its instability, acetolactic acid had to be produced *in situ* from pyruvic acid using *bs*ALS (2 pyruvic acid → 1 acetolactic acid + 1 CO_2). The K_m given refers to the experimentally determined K_m based on pyruvic acid input divided by the stoichiometric factor of two. Based on previous experiments, we herein assume 100% conversion and selectivity for acetolactic acid production from pyruvic acid.

Supplementary Table 4. Activity of purchased enzymes alcohol oxidase (AOX), catalase (CAT), glutamate dehydrogenase (GluDH) and formate dehydrogenase (FDH).

Enzyme	Activity	Reference
**AOX**	10 - 40 U mg^{-1}	Sigma-Aldrich (A2404)
**CAT**	≥500000 U mL^{-1}	Sigma-Aldrich (02071)
**GluDH**	≥35 U mg^{-1}	Sigma-Aldrich (G2626)
**FDH**	~2 U mg^{-1}	Megazyme (E-FDHCB)

Supplementary Table 5. Enzyme kinetics derived from previous publications of enzymes used in the cascades.

Enzyme	K_m [mM]	Activity k_{cat} (s^{-1})	Reference
**FLS**	34.14	0.1457	S13
**FLSM3**	23.59	0.2375	S13
**GALS**	59	0.43	S3
***pp*D-KDPD**	0.35 ± 0.01	75.9 ± 0.6	S9
***pp*KGSALDH**	0.10 ± 0.01	31.7	S10
***ec*PROC**	0.139		S14

Supplementary Table 6. Concentration of enzymes in glycine and serine cascade from glycolaldehyde with optimized parameters for glycine cascade (glycine opt). Enzyme concentration of *ta*ALDH variant M42, glycolate oxidase (GLOX), alanine dehydrogenase (AlaDH), unspecific threonine aldolase (LTA) and catalase (CAT) used in the cascade.

	**Glycine**	**Glycine opt**	**Serine**
**Core enzyme**	**Conc. [mg mL^{-1}]**	**Conc. [mg mL $^{-1}$]**	**Conc. [mg mL^{-1}]**
**M42**	2.26	2.26	2.26
**GLOX**	0.18	0.18	0.18
**AlaDH**	0.01	0.23	0.23
**Pathway dependent enzyme**	**Conc. [mg mL^{-1}]**	**Conc. [mg mL^{-1}]**	**Conc. [mg mL^{-1}]**
**LTA**			1.72
**Additional enzyme**	**Units [µmol min^{-1}]**	**Units [µmol min^{-1}]**	**Units [µmol min^{-1}]**
**CAT**	**350**	**350**	**350**

Supplementary Table 7. Concentration of enzymes in L-aspartic acid, L-valine, L-glutamic acid and L-proline cascade from dihydroxyacetone (L-aspartic acid and L-valine) and dihydroxyacetone and glycolaldehyde (L-glutamic acid and L-proline) with optimized parameters. Black concentrations are mg mL^{-1}, grey concentrations µg mL^{-1}.

	L-Aspartic acid	L-Aspartic acid opt	L-Valine	L-Valine opt	L-Glutamic acid	L-Glutamic acid opt	L-Proline	L-Proline opt
**Core enzyme**	**Conc. [mg mL^{-1}]**		**Conc. [mg mL^{-1}]**		**Conc. [mg mL^{-1}]**		**Conc. [mg mL^{-1}]**	
**DHAK**	3.30		3.30		3.30		3.30	
**TPI**	0.03		0.03		0.03		0.03	
**GAPN**	3.00		3.00		3.00		3.00	
**PGM**	2.55		2.55		2.55		2.55	
**ENO**	0.30		0.30		0.30		0.30	
**PYK**			4.10		4.10		4.10	
**Pathway dependent enzyme**	**Conc. [µg mL^{-1}]**	**Conc. [µg mL^{-1}]**	**Conc. [µg mL^{-1}]**	**Conc. [µg mL^{-1}]**	**Conc. [µg mL^{-1}]**	**Conc. [µg mL^{-1}]**	**Conc. [µg mL^{-1}]**	**Conc. [µg mL^{-1}]**
**PEPCK**	15.68	15.68						
**AspDH**	12.80	12.80						
**ALS**			109.88	109.88				
**KARI**			2250.36	11251.80				
**DHAD**			1126.00	1126.00				
**LeuDH**			115.20	115.20			3000.00	600
**KDGA**					363.00	1815.00	363.00	1815.00
**D-KDPD**					17.64	17.64	17.64	17.64
**L-KDPD**					74.00	74.00	74.00	74.00
**KGSALDH**					90.00	90.00	90.00	90.00
**L-GluDH**					22.50	22.50		
**NOX**					1.92	1.92		

**PROC**	20.00	20.00
**FDH**	75.00	75.00

Supplementary Table 8. Concentration of enzymes of the methanol to glycolaldehyde or methanol to dihydroxyacetone module of the corresponding amino acid cascade. Glycolaldehyde synthase (GALS) was used in glycine and serine system. Formolase-M3 (FLSM3) was used in L-aspartic acid and L-valine system. Formolase (FLS) was used in L-glutamic acid and L-proline system. Alcohol oxidase (AOX) and catalase (CAT) in grey were used in all systems.

	Glycine	Serine	L-Aspartic acid	L-Valine	L-Glutamic acid	L-Proline
**Methanol enzyme**	**Conc. [mg mL^{-1}]**					
**GALS**	20					
**FLSM3**			10			
**FLS**					10	
**AOX**	0.2					
**Additional enzyme**	**Units [µmol min^{-1}]**					
**CAT**	**350**					

Supplementary Table 9. Time course of metabolites of the L-aspartic acid cascade upscaled to a total reaction volume of 15 mL as measured *via* HILIC LC-MS. Step one refers to the conversion of CO_2-derived methanol *via* formaldehyde to dihydroxyacetone. Step two represents the subsequent production of L-aspartic acid from the dihydroxyacetone produced *in situ*. Metabolomics was conducted as single measurement (n = 1 sample). Expectedly, measurement of the negative control for every individual time point did generally not give any signal and is thus not shown. The isomers 3-phosphoglyceric acid and 2-phosphoglyceric acid could hardly be separated by LC-MS given their very similar retention times. The values below therefore only reflect trends. Measurement of L-alanine and pyruvic acid served the elucidation of side product formation. HILIC LC-MS measurements of L-aspartic acid were consistent with quantification of this compound by HPLC. Oxaloacetic acid was not found in any experimental sample, though its standard could not be detected either. Abbreviations: DHAP, dihydroxyacetone phosphate. GA3P, glyceraldehyde 3-phosphate. 3-PG, 3-phosphoglyceric acid. 2-PG, 2-phosphoglyceric acid. PEP, phosphoenolpyruvic acid. L-Asp, L-aspartic acid. L-Ala, L-alanine. Source data for this table are provided as a Source Data file.

	**Step 1**			**Step 2**							
	**0.0 h**	**5.0 h**	**25.0 h**	**25.25 h**	**25.5 h**	**26.0 h**	**27.0 h**	**28.0 h**	**30.0 h**	**40.0 h**	**45.0 h**
**DHAP**	no signal (< 1 mM)										
**GA3P**	no signal (< 2.5 mM)										
**3-PG**	<< 2.5 mM	no signal	<< 2.5 mM	no signal	<< 2.5 mM	<< 2.5 mM	<< 2.5 mM	<< 2.5 mM	<< 2.5 mM	<< 2.5 mM	no signal
**2-PG**	no signal	no signal	no signal	no signal	763.61 µM	585.49 µM	573.96 µM	419.96 µM	381.51 µM	no signal	no signal
**PEP**	no signal	no signal	no signal	no signal	< 1 mM	< 1 mM	< 1 mM	< 1 mM	< 1 mM	no signal	no signal
**L-Asp**	no signal	no signal	no signal	no signal	< 1 mM	< 1 mM	> 1 mM	> 1 mM	> 1 mM	> 1 mM	> 1 mM
**L-Ala**	no signal (< 1 mM)										
**Pyruvic acid**	no signal	<< 1 mM	<< 1 mM	<< 1 mM	< 1 mM	< 1 mM	< 1 mM	< 1 mM	< 1 mM	< 1 mM	< 1 mM

Supplementary Table 10. Solar performance and energy production metrics for agricultural and energy systems across different regions (represented by Northern Denmark and Central Queensland Australia), with focus on methanol, alanine, and valine production compared to cropy yields.

Solar Performance Metrics	Average	Northern Denmark	Central Queensland	Unit	Reference
**Direct average solar irridation**	1600	1200	2200	kWh/m²/year	S15
**Solar panel efficiency**	20%	20%	20%		
**Coverage of area (e.g. Agri-Photovoltaic)**	50%	50%	50%		S16
**Total energy output for 1 ha p.a.**	1600	1200	2200	MWh/ha/year	
**Energy required for:**					
**Hydrogen production**			50	MWh per to	S17
**CO_2 air capture**			1	MWh per to	S18
**Synthesis of methanol**			11	MWh per to	*
**Synthesis of Ammonia**			9	MWh per to	**
**Synthesis of Alanine**			14	MWh per to	***
**Synthesis of Valine**			20	MWh per to	****
**Amount to be produced from 1 ha:**					
**Methanol**	145	109	200	to/ha/year	
**Alanine**	114	86	157	to/ha/year	
**Valine**	80	60	110	to/ha/year	

Amount of soy (whole plant): Australia < 2 to/ha/ year (average over the last 15 years) [S19]. Amount of wheat (whole plant): Poland (as reference to Denmark) < 5 to/ha/year [S20].

* 1,75 to of CO_2 and 0,1875 to of Hydrogen per to methanol, no heat utilization, numbers rounded, ** 0.177 to of Hydrogen per to of Ammonia, no utilization of excess heat, *** 1 to Alanine from 0,93 to of methanol and 0,19 to of NH3 => 11,94 MWh per to, **** 1 to Valine from 1,64 to of methanol and 0,145 to of NH3 => 19,4 MWh per to

Supplementary Table 11. New synthetic gene sequences used in this study. Glycolaldehyde synthase sequence is based on Lu et al.[S3] but slightly modified for our cloning procedure.

Gene	Sequence
**Glycolaldehyde synthase (GALS)**	ATGCATCATCATCATCATCAtCTCGACatgGCTTCTGTTCACGGTACCACCTACGAACTGCTGCGTCGTCAGGGTAT<br>CGACACCGTTTTCGGTAACCCGGGTTCTAACGAACTGCCGTTCCTGAAAGACTTCCCGGAAGACTTCCGTTACAT<br>CCTGGCTCTGCAGGAAGCTTGCGTTGTTGGTATCGCTGACGGTTACGCTCAGGCTTCTCGTAAACCGGCTTTCAT<br>CAACCTGCACTCTGCTGCTGGTACCGGTAACGCTATGGGTGCTCTGTCTAACGCTCGTACCTCTCACTCTCCGCT<br>GATCGTTACCGCTGGTCAGCAGACCCGTGCTATGATCGGTGTTGAAGCTGGGGAAACCAACGTTGACGCTGCTA<br>ACCTGCCGCGTCCGCTGGTTAAATGGTCTTACGAACCGGCTTCTGCTGCTGAAGTTCCGCACGCTATGTCTCGTG<br>CTATCCACATGGCTTCTATGGCTCCGCAGGGTCCGGTTTACCTGTCTGTTCCGTACGACGACTGGGACAAAGACG<br>CTGACCCGCAGTCTCACCACCTGTTCGACCGTCACGTTTCTTCTTCTGTTCGTCTGAACGACCAGGACCTGGACA<br>TCCTGGTTAAAGCTCTGAACTCTGCTTCTAACCCGGCTATCGTTCTGGGTCCGGACGTTGACGCTGCTAATGCTA<br>ACGCTGACTGCGTTATGCTGGCTGAACGTCTGAAAGCTCCGGTTTGGGTTGCTCCGTCTGCTCCGCGTTGCCCG<br>TTCCCGACCCGTCACCCGTGCTTCCGTGGTCTGATGCCGGCTGGTATCGCTGCTATCTCTCAGCTGCTGGAAGG<br>TCACGACGTTGTTCTGGTTATCGGTGCTCCGGTTTTCCGTTACGTTTTTTACGACCCGGGTCAGTACCTGAAACCG<br>GGTACCCGTCTGATCTCTGTTACCTGCGACCCGCTGGAAGCTGCTCGTGCTCCGATGGGTGACGCTATCGTTGC<br>TGACATCGGTGCTATGGCTTCTGCTCTGGCTAACCTGGTTGAAGAATCTTCTCGTCAGCTGCCGACCGCTGCTCC<br>GGAACCGGCTAAAGTTGACCAGGACGCTGGTCGTCTGCACCCGGAAACCGTTTTCGACACCCTGAACGACATGG<br>CTCCGGAAAACGCTATCTACCTGAACGAATCTACCTCTACCACCGCTCAGATGTGGCAGCGTCTGAACATGCGTA<br>ACCCGGGTTCTTACTACTTCTGCGCTGCTGGTGGTCTGGGTTTCGCTCTGCCGGCTGCTATCGGTGTTCAGCTGG<br>CTGAACCGGAACGTCAGGTTATCGCTGTTATCGGTGACGGTTCTGCTAACTACTCTATCTCTGCTCTGTGGACCG<br>CTGCTCAGTACAACATCCCGACCATCTTCGTTATCATGAACAACGGTACCTACGGTATGCTGCGTTGGTTCGCTG<br>GTGTTCTGGAAGCTGAAAACGTTCCGGGTCTGGACGTTCCGGGTATCGACTTCCGTGCTCTGGCTAAAGGTTACG<br>GTGTTCAGGCTCTGAAAGCTGACAACCTGGAACAGCTGAAAGGTTCTCTGCAGGAAGCTCTGTCTGCTAAAGGTC<br>CGGTTCTGATCGAAGTTTCTACCGTTTCTCCGGTTAAATAA
**Glycolate oxidase (GLOX)**	ATGGATGGAAATTACCAACGTGAATGAATATGAAGCGATCGCCAAACAGAAACTGCCGAAAATGGTGTATGATTAT<br>TATGCAAGCGGTGCCGAAGATCAGTGGACCCTGGCAGAAAATCGTAATGCATTTAGCCGTATTCTGTTTCGTCCG<br>CGTATTCTGATTGATGTGACCAATATTGATATGACCACCACCATTCTGGGCTTCAAAATTAGCATGCCGATTATGAT<br>TGCACCGACCGCAATGCAGAAAATGGCACATCCGGAAGGTGAATATGCAACCGCACGTGCAGCAAGCGCAGCAG<br>GCACCATTATGACCCTGAGCAGCTGGGCAACCAGCAGCGTTGAAGAAGTTGCAAGCACCGGTCCGGGTATTCGT<br>TTTTTTCAGCTGTATGTGTATAAAGACCGTAATGTTGTTGCACAGCTGGTTCGTCGTGCAGAACGTGCAGGTTTTA<br>AAGCAATTGCACTGACCGTTGATACACCGCGTCTGGGTCGTCGTGAAGCAGATATCAAAAATCGTTTTGTTCTGC<br>CTCCGTTTCTGACCCTGAAAAACTTTGAAGGTATTGACCTGGGCAAAATGGATAAAGCAAATGATAGCGGTCTGA<br>GCAGTTATGTTGCAGGTCAGATTGATCGTAGCCTGAGCTGGAAAGATGTTGCATGGCTGCAGACCATTACCAGCC<br>TGCCGATTCTGGTTAAAGGTGTTATTACCGCAGAAGATGCACGTCTGGCAGTTCAGCATGGTGCAGCAGGTATTA<br>TTGTTAGCAATCATGGTGCACGTCAGCTGGATTATGTTCCGGCAACCATTATGGCACTGGAAGAAGTGGTTAAAG<br>CAGCACAGGGTCGTATTCCGGTTTTTCTGGATGGTGGTGTGCGTCGTGGCACCGATGTTTTTAAAGCCCTGGCAC<br>TGGGTGCAGCCGGTGTTTTTATTGGTCGTCCGGTTGTTTTTAGCCTGGCAGCCGAAGGTGAAGCGGGTGTTAAAA<br>AAGTTCTGCAGATGATGCGTGATGAATTTGAACTGACAATGGCACTGAGCGGTTGTCGTAGTCTGAAAGAAATTA<br>GCCGTAGCCATATTGCAGCAGATTGGGATGGTCCGAGCAGCCGTGCAGTTGCCCGTCTG
**(Non)-phosphorylative glyceraldehyde 3-phosphate dehydrogenase (GAPN)**	AAAAAACATATGGTTGAGCCCTTCGTCCCCGAGGGGGAAATATTCGAAGGAATCTTCCGGCAGAACGAGGGTATA<br>CCCGAATTCGCAACGTACGTAAACGGGGAGTGGGTGTTTACCGGGAAGACAGCGGAGGTAAGAAGCCCGATAGA<br>CGGCTCGCTGATAGCTAGGGTAAGCCTGAGCGATATGGCCCTTTCGAACCGGGCGGTGGCGGCGGCATATTCC<br>GCTGGGAGGCATGAGATAAGGGACACGCCGGGTGAAAAGAGGTTGGAGGCTTTTTTAAAGGTGGCGGAGCTTAT<br>TAGGGACTCATTCGACGATTTCGTCACCGCCCTGGTGCTCGACGCCGGCAAGCCGCTTTCAAACGCCCGCGGTG<br>AGGTTACCGCCACAATCGAGAGACTTGAGAAGACGACGATGGAGTTCGGCAGGTTGATAGGGGACTACATTCCC<br>GGCGACTGGAGCGCAGAAAGCCTTGGAAGCGAGGGGATAGTCAAGAGGGAGCCCTACGGGGTTGTGCTCGCAA<br>TAAGTCCCTACAACTACCCGCTGTTTATCTCCACCGCCAAGATCGTTCCGGCCCTACTTGCCGGAAACGCCGTGC<br>TTCTGAAGCCATCTTCCCAGGATCCCCTCGCTCCCCTACTCCTGTCAAGGGTTCTTCAGCTTGCGGGAATTCCTG<br>AAAGCGCCTATCACCTTCTAACTGTCCCAGGAGCGCTGATGGACTCCATACTGGCCGACAGAAGAATAAGGGCG<br>GTCACGTTTACGGGCAGCACCGAGGTCGGCGAGCACATCCTATCCATGGGAGGTATAAAGTTCTACCACATGGA<br>ACTCGGTGGTAAGGACCCAGCGGTGGTTCTGGACGACGCTCCGCTCGAAGAAACCGTGGAAAAGCTTGTCAAAG<br>GTATGGTGAGCTACTCCGGCCAGAGGTGCGATGCGATAAGGCTAATCATCGCCGAGGAGGGGATATACGAGCAG<br>CTTAAGAGAGAACTCGTGGCTGCCCTCTCAAAAATAGAGCCCGAGAACCCGCTTGAAGACGAGGACGCCATTAT<br>GGGGCCACTGATAAACGAGCGGAGCGCTGAAAAAATAGAAGAGGTTTACAGGGATGCCCTGGAGAAGGGAGCG<br>GTGCCCCTAACGGGGTTCAAGAGGAAAGGAGCGTACGTATGGCCTGTTCTCCTGGAGGCCAGCAGGGAAGTTCT<br>GCCTGGACTCAGGGCGTTCCAGGAGGATGTTTTCGGTCCGCTCACAATCCTGGTTAAGGTTTCGAACGAGGACG<br>AGGCCGTTGAGCTTGCCAACTCATCGAGGTTTGGCCTGGACGCCGCAGTTTTCAGTGGGGACGATTCCAGGGCG<br>AGGAAAGTCGCGAGGAGGCTTGAAGTCGGGGCCGTCTTCATAAACGAGTTTCCACGGCACGGGATCGGTTATTA<br>CCCCTTCGGGGGCATGAAAGACAGCGGCATCGGAAGGGAGGGCATAGGCTATTCCATCGAGACCCTCACTACGA<br>CTAAGACGATCGTCAGGAACTATCGGGGAAGGGGTGTCTGGGACTACATCTGACTCGAGAAAAAA

Supplementary Table 12. Description and settings for data analysis procedure of HILIC LC-MS metabolic profiling experiments of the upscaled L-valine and L-aspartic acid cascades *via* MassHunter ProFinder software from Agilent Technologies following the Wizard Batch targeted feature extraction workflow.

Step	Sub-step	Parameter	Description
**Formula targets**	Formula Source	Database	Refer to Supplementary table 13
		Matches per formula	Maximum number of matches: 1
		Values to match	Mass and retention time (retention time optional)
	Ion species	Allowed ion species	Negative: -H
		Charge states, if not known	Charge state range: 1
	Charge state	Isotope grouping, isotope model	Common organic (no halogens)
		Charge state	Limit assigned charge states to a range of: 1-2
**Matching tolerances and scoring**	Formula matching	Match tolerance	Masses: ± 10.00 ppm
			Retention times: ± 0.300 minutes
		Expansion of values for chromatogram extraction	Possible m/z: symmetric (ppm): ± 35.0
			Limit EIC extraction range
			Expected retention time: symmetric: ± 1.50 minutes
	Scoring	Contribution to overall score	Mass score: 100.00
			Isotope abundance score: 60.00
			Isotope spacing score: 50.00
			Retention time score: 0.00
		Expected data variation	MS mass: 2.0 mDa + 5.6 ppm
			MS isotope abundance: 7.5%
			MS/MS mass: 5.0 mDa + 7.5 ppm
			Retention time: 0.115 min
	Result filters	Matching criteria, low score matches	Matches for which the overall score is low: warn if score is < 75.00
		Matching criteria, single ion matches	Matches for which only a single evidence ion is observed, but a second evidence ion is predicted from the formula. Warn if the (unobserved) second ion´s abundance is expected to be > 50.00
**EIC peak integration and filtering**	Integration	Integrator selection	Agile 2
	Smoothing	Chromatogram smoothing	Smoothing function: Gaussian
			Function width: 9 points
			Gaussian width: 5.000 points
	Peak filters	Filter on	Peak height
		Height filters	Absolute height ≥ 1000 counts
		Maximum number of peaks	Limit (by height) to the largest: 5
	Chromatogram format	Chromatogram data format	Centroid when available, otherwise Profile
**Spectrum extraction and centroiding**	Peak spectrum	Spectra to include	Average scans > 10% of peak height
		TOF spectra	Exclude if above 20.0% of saturation, in the m/z ranges used in the chromatogram
			Never return an empty spectrum
		Peak spectrum background	MS: Current background spectrum
	Centroiding	Peak location	Maximum spike width: 2
			Required valley: 0.70
	Spectrum format	Mass spectral data format	Centroid when available, otherwise Profile
**Post-processing filters**	Find by formula filters	Score (Tgt)	≥ 70.00
	Minimum filter matches	A compound must satisfy the checked Find by Formula filter conditions in at least 1 file (for unicum, n = 1 sample)	In at least one sample group

Supplementary Table 13. Target list of compounds for metabolic profiling *via* HILIC LC-MS.

Compound	Formula	Rt [min]	Monoisotopic mass [u]	Conc. [µM]
**Dihydroxyacetone phosphate**	$C_3H_7O_6P$	/	169.9980	200
**Glyceraldehyde 3-phosphate**	$C_3H_7O_6P$	/	169.9980	200; 500 - 7500
**3-Phosphoglyceric acid**	$C_3H_7O_7P$	20.0	185.9929	200; 500 - 7500
**2-Phosphoglyceric acid**	$C_3H_7O_7P$	19.7	185.9929	50 - 1000
**Phosphoenolpyruvic acid**	$C_3H_5O_6P$	20.7	167.9824	200 - 500
**Pyruvic acid**	$C_3H_4O_3$	8.8	88.016	0.2 - 500
**Acetolactic acid**	$C_5H_8O_4$	/	132.0423	/
**2,3-Dihydroxyisovaleric acid**	$C_5H_{10}O_4$	7.5	134.0579	0.2 - 500
**2-Ketoisovaleric acid**	$C_5O_3H_8$	5.8	116.0473	0.2 - 500
**L-Valine**	$C_5H_{11}NO_2$	10.5	117.079	200
**Oxaloacetic acid**	$C_4H_4O_5$	/	132.0059	200
**L-Aspartic acid**	$C_4H_7NO_4$	16.3	133.0375	200
**L-Alanine**	$C_3H_7NO_2$	12.9	89.0477	200
**Acetoin**	$C_4H_8O_2$	/	88.0523	/
**α-Aminobutyric acid (internal standard)**	$C_4H_9NO_2$	11.7	103.0633	/

Supplementary references

1. Güner, S., Wegat, V., Pick, A. & Sieber, V. Design of a synthetic enzyme cascade for the in vitro fixation of a C 1 carbon source to a functional C 4 sugar. *Green Chemistry* (2021).
2. Willers, V.P., Döring, M., Beer, B. & Sieber, V. Cell-free enzymatic L-alanine synthesis from green methanol. *Chem Catalysis* **3**, 100502 (2023).
3. Lu, X. et al. Constructing a synthetic pathway for acetyl-coenzyme A from one-carbon through enzyme design. *Nature communications* **10**, 1-10 (2019).
4. Gmelch, T.J., Sperl, J.M. & Sieber, V. Molecular dynamics analysis of a rationally designed aldehyde dehydrogenase gives insights into improved activity for the non-native cofactor NAD+. *ACS Synthetic Biology* **9**, 920-929 (2020).
5. Guterl, J.K. et al. Cell-free metabolic engineering: production of chemicals by minimized reaction cascades. *ChemSusChem* **5**, 2165-2172 (2012).
6. Willers, V.P., Beer, B. & Sieber, V. Integrating Carbohydrate and C1 Utilization for Chemicals Production. *ChemSusChem* **16**, e202202122 (2023).
7. Sutiono, S., Teshima, M., Beer, B., Schenk, G. & Sieber, V. Enabling the direct enzymatic dehydration of D-glycerate to pyruvate as the key step in synthetic enzyme cascades used in the cell-free production of fine chemicals. *ACS Catalysis* **10**, 3110-3118 (2020).
8. Gmelch, T.J., Sperl, J.M. & Sieber, V. Optimization of a reduced enzymatic reaction cascade for the production of L-alanine. *Scientific reports* **9**, 1-9 (2019).
9. Sutiono, S., Siebers, B. & Sieber, V. Characterization of highly active 2-keto-3-deoxy-L-arabinonate and 2-keto-3-deoxy-D-xylonate dehydratases in terms of the biotransformation of hemicellulose sugars to chemicals. *Applied Microbiology and Biotechnology* **104**, 7023-7035 (2020).
10. Beer, B., Pick, A. & Sieber, V. In vitro metabolic engineering for the production of α-ketoglutarate. *Metab. Eng.* **40**, 5-13 (2017).
11. Nowak, C. et al. A water-forming NADH oxidase from Lactobacillus pentosus suitable for the regeneration of synthetic biomimetic cofactors. *Front. Microbiol.* **6** (2015).
12. Beber, M.E. et al. eQuilibrator 3.0: a database solution for thermodynamic constant estimation. *Nucleic Acids Research* **50**, D603-D609 (2021).
13. Cai, T. et al. Cell-free chemoenzymatic starch synthesis from carbon dioxide. *Science* **373**, 1523-1527 (2021).
14. Rossi, J.J., Vender, J., Berg, C.M. & Coleman, W.H. Partial purification and some properties of delta1-pyrroline-5-carboxylate reductase from Escherichia coli. *J. Bacteriol.* **129**, 108-114 (1977).
15. Data obtained from the “Global Solar Atlas 2.0, a free, web-based application is developed and operated by the company Solargis s.r.o. on behalf of the World Bank Group, utilizing Solargis data, with funding provided by the Energy Sector Management Assistance Program (ESMAP). For additional information: https://globalsolaratlas.info
16. Jacob Stid, S.S., Anthony Kendall et al. From Fields to Photovoltaics: Effects of Agrisolar Co-Location on Food, Energy, Water, and Economic Security. *PREPRINT (Version 1) available at Research Square* (2023).
17. Stojić, D.L., Marčeta, M.P., Sovilj, S.P. & Miljanić, Š.S. Hydrogen generation from water electrolysis—possibilities of energy saving. *Journal of Power Sources* **118**, 315-319 (2003).
18. Kiani, A., Jiang, K. & Feron, P. Techno-Economic Assessment for CO2 Capture From Air Using a Conventional Liquid-Based Absorption Process. *Frontiers in Energy Research* **8** (2020).
19. ABARES. “Yield of soybeans in Australia from financial year 2010 to 2025 (in metric tons per hectare).” Chart. September 24, 2024. Statista. Accessed February 16, 2025. https://www.statista.com/statistics/631890/australia-soybean-yield/
20. Central Statistical Office of Poland. (December 23, 2024). Cereals yields in Poland from 2010 to 2023, by type (in quintals per one hectare). In Statista. Retrieved

February 16, 2025, from https://www.statista.com/statistics/1129640/poland-cereals-yields-by-type/
