# Supplementary material for: Plug-and-play – enzymatic amino acid production from methanol and carbon dioxide
Source: Nat Commun. 2026 Jun 17;17:5363. doi: 10.1038/s41467-026-74522-x (PMC13276030; doi:10.1038/s41467-026-74522-x)
Supplement: Supplementary file 2 — Description of Additional Supplementary Files [file 41467_2026_74522_MOESM2_ESM.pdf]

Description of Additional Supplementary Files

File Name: Supplementary Data 1
Description: DNA sequences of all proteins expressed, purified and used in this work.

File Name: Supplementary Data 2
Description: ChemDraw file for Figure 1b showing all metabolic routes for the production of the six amino acids.
