# Supplementary material for: Plug-and-play – enzymatic amino acid production from methanol and carbon dioxide
Source: Nat Commun. 2026 Jun 17;17:5363. doi: 10.1038/s41467-026-74522-x (PMC13276030; doi:10.1038/s41467-026-74522-x)
Supplement: Supplementary file 6 — Transparent Peer Review file [file 41467_2026_74522_MOESM6_ESM.pdf]

nature portfolio

Peer Review File

Plug and Play – Enzymatic Amino Acid Production from Methanol and Carbon Dioxide

Corresponding Author: Professor Volker Sieber

This file contains all reviewer reports in order by version, followed by all author rebuttals in order by version.

Version 0:

Reviewer comments:

Reviewer #1

(Remarks to the Author)
This work presents the production of six amino acids from methanol or co2-derived methanol via a set of enzymatic pathways. The pathway design is great and involves several so-called plug-and-play modules.. The synthesis of all six amino acids is demonstrated with high conversion yield, at least for some of them. Generally, it meets the novelty and the significance standard of the journal and can be published after major revisions.
-The manuscript format, writing typos, data presenting are suggested to be examined carefully and improved.
-The introduction is suggested to be further refined. Its current format reads like a report rather than a scientific paper.
-The hexagon in Fig 1 is kind of misleading. It looks like a phenyl group. It may be changed to cycle or sth else.
-The Fig 2C and Fig 3 could be refined with more details or in another more-straightforward format. Fig 2C uses 10 mM C2 or C3 substrates reacted for 5 h, which Fig 3 uses 30 mM methanol for 24 h. In the text, those data are grouped based on the amino acid type and reported together. It is just kind of not very reader-friendly.
-The data presenting could be further optimized. For example, the Fig S10 is abrupt. Why it only presents the alanine as the byproduct for the synthesis of proline? Why not showing other byproducts under other conditions? Indeed, as the production of many amino acids are demonstrated, the byproduct issue should be particularly discussed for those involved.
-The legend in the Fig 4 seems not right. The color shown is not the same as the legend describes. Besides, in Fig 4B, why the DHA is not ~220 mM C at the point of 25 h ? It is only ~105 mM C based on the figure.
-For valine production from CO2-derived methanol, as the data shown in Fig 4, why the conversion yield (37%) is much lower than those from methanol directly (78%)? Why the conversion almost stops at 12 h?
-A table summarizing all the conversion yields of those amino acids is suggested. It can also contains the comparison with other technologies of in vitro amino acids synthesis from C1 sources, or highlights the advantages of the method used in this present work.
-Fig 5 is unnecessary. Please remove it.

Reviewer #2

(Remarks to the Author)
This study presents a multi-enzyme cascade system design for synthesizing a series of amino acids from methanol, CO_2, and ammonia. Through modular replacement and enzymatic adjustments, the authors successfully achieved the synthesis of amino acids with carbon chain lengths ranging from C2 to C6. The work constitutes a notable achievement in demonstrating in vitro enzymatic synthesis of amino acids, offering a well-executed case study for modular biocatalytic systems. The systematic approach to pathway engineering and module optimization provides valuable insights for synthetic biology applications in value-added chemical production.
However, the data presented in the manuscript lack systematic organization, and the optimization process of the multi-enzyme catalytic system is described in an overly cursory manner. Critical scientific inquiries into the roles of key enzymes and pivotal catalytic steps remain insufficiently explored. Furthermore, the schematic depiction of the amino acid synthesis pathway lacks clarity in crucial mechanistic details. The authors are advised to comprehensively refine the manuscript by strengthening experimental rigor, elaborating on enzyme screening/engineering strategies, clarifying pathway design principles, and providing in-depth mechanistic discussions. The revised manuscript should be resubmitted for reevaluation after addressing these fundamental issues.
The authors are strongly advised to address the following critical aspects in their revision:

1.The step-by-step optimization workflow should be systematically elaborated, including enzyme ratio adjustment and screening of reaction conditions (pH, temperature, cofactor levels), to clarify how to maximize catalytic efficiency and pathway yield.
2.Experimental data supporting the selection and engineering of pivotal enzymes (e.g., carbon chain elongation enzymes, ammonia assimilation enzymes) must be supplemented.
3.The inhibitory effects of elevated methanol concentrations on amino acid carbon yield require systematic investigation. The authors are strongly advised to optimize the reaction system under high substrate-loading conditions to mitigate metabolic flux bottlenecks and enhance product titer.
4.The methanol concentration-dependent alteration of the partitioning of the C2/C3 products of FLS-in particular the ratio of DHA to glycolaldehyde and its cascading effect on the glutamate/proline biosynthetic pathway remain mechanistically unresolved. This critical metabolic bifurcation node needs to be studied rigorously by performing.

Reviewer #3

(Remarks to the Author)
Aminoacids are vital components of the food chain. Carbon dioxide is supposed to be the impornat source of C1 products in the future. Enzymatic catalysis and heterogeneous catalysis are known to have advantages and disadvantages, which may be the barriers for the potential commercialization. The paper is focused on the inventive combination of heterogeneous catalysis aiming at the production of methanol by CO2 hydrogenation and catalysis by enzymes as a downstream conversion of methanol produced at the first stage into valuable aminoacids. The authors presented this strategy by using not just one example of an aminoacid but the impressive range of amino acids with a different number of carbon atoms. Furtheremore, the authors tried to estimate the potential of this new technology for tow geographic regions and showed the applicability of the cascade technology. The paper seems to be a significant contribution to the fields of CO2 utilization and artificial food, especially produced from a waste. The following comments should be taken into account in the revision of the manuscript:
- The authors considered (and tested) a commercial catalyst, which provides a low efficiency in CO2 hydrogenation. Now new catalysts have been reported with a CO2 conversion reaching 25-30% and the optimal temperature of the process was found to be close to 250oC. Therefore, the authors may correct the figures to show a better performance of the cascade technology and improved economics/energetics.

Reviewer #4

(Remarks to the Author)
NCOMMS-25-29042-T

Plug and Play – Enzymatic Amino acid production from Methanol and Carbon dioxide
This study describes one-pot enzymatic systems for synthesizing serine, L-aspartic acid, L-valine, L-glutamic acid, and L-proline, form methanol, ammonia, and carbon dioxide. However, the reported titers for the abovementioned amino acids are relatively low ranging from 1-10 mM. The manuscript has enormous data, but it was poorly organized.
Major issue:
1. The reported titers for the amino acids are relatively low ranging from 1-10 mM. How is it compared to engineered cells to produce amino acids from methanol via metabolic engineering approaches? For instance, metabolic engineering of methanol-utilizing host such as Pichia pastoris will be a straightforward method, and it will simplify the enzyme purification process that will significantly reduce the preparation cost.
2. All the enzyme parts used in this study are previously reported. There is no gain-of-new-information, which leads to the poor novelty. The authors have previously published a Chem Catalysis paper on alanine production from methanol. It seems to me that it is a simple expansion of the same design to several more amino acids.
3. The authors are encouraged to scale-up 1-2 amnio acid biosynthesis system, and provide more evidence that the enzymatic approach has advantage in turns of productivity and cost, compared to conventional engineered microbes via metabolic engineering. At this moment, all the reactions are only carried out at 100-200 µl. Can the authors demonstrate a 5-10 L reaction volume to provide scalability evidence?
4. If the biosynthetic pathways are not cofactor (NADPH, NADH, ATP) neutral, how is the cost of replenishing these cofactors?
5. Metabolite profiling should be provided during the course the reaction, to monitor the intermediate levels. These data should be provided in the SI.
6. The figures in the maintext are poorly presented. Some of the biosynthetic routes can be organized in a better way, and combined with the corresponding data in the maintext.
7. Since the serine production is relatively low, it is recommended to move the data to the SI.
Minor issues:
1. The enzymes and genes should be formatted in a uniform way. For instance, ltaE or ltAE.
2. All strains should be full name at the first appearance, and as abbreviated forms thereafter. For instance, line 450-460. (ATCC 7953) should not be an italicized form.
3. All restriction enzymes should have the first 3-letters in the italicized form (BsaI).
4. There are numerous grammar errors in the maintext and SI. Many of chemicals should be in the capital size.

Version 1:

Reviewer comments:

Reviewer #1

(Remarks to the Author)
The authors have addressed most of my previous concerns. However, the questions and suggestions from review 4 were not addressed well in my opinion.

Moreover, I feel that the “plug-and-play” of the multienzyme system was not well featured. For example, several enzymes should be grouped into one module, and several for the other. How these different modules could be organized and how the adaptation issue could be solved?

Also, it might be noticed that in these months, several works on cell-free multienzymatic synthesis of CO2-derived glycine and aspartate have come out (https://advanced.onlinelibrary.wiley.com/doi/10.1002/adsc.70238; https://pubs.acs.org/doi/10.1021/acssuschemeng.5c11185), which would hurt the novelty of this work..

Reviewer #2

(Remarks to the Author)
The author has provided a satisfactory answer to my question; I recommend accepting it.

Reviewer #3

(Remarks to the Author)
The authors revised the paper by addressing all the comments. Now the paper can be accepted for publication

Reviewer #4

(Remarks to the Author)
NCOMMS-25-29042A

Plug and Play – Enzymatic Amino acid production from Methanol and Carbon dioxide
This study describes one-pot enzymatic systems for synthesizing serine, L-aspartic acid, L-valine, L-glutamic acid, and L-proline, form methanol, ammonia, and carbon dioxide. But the overall manuscript is still poorly organized after revision. It is difficult for non-expert to follow the logic. Although the metabolic routes for glycine and serine lack novelty, I found that the design of metabolic routes for L-glutamate and L-proline is very elegant and insightful. The manuscript might focus more on the key findings on L-glutamate and L-proline production from CO2. The revision partially addressed my concerns, but there are still more improvements required. I personally did not advocate using purified enzymes for amino acid synthesis as the cost of enzymes might be higher than the product itself. Nevertheless, this work might have some insights that would inspire others in the field.
Major issue:
1. As shown in Figure S1 and S2, these figures can be combined together since glycine and serine share the same pathway of glycolate as the intermediate. The combined metabolic route can be supplemented in the maintext, followed by all the corresponding data to support (all the main findings, with Estimation of reaction Gibbs energy). By doing so, it will favor the readers to better understand the concept of “Plug and Play” and modular design. (a potential new Figure 2)
2. Figure S3/S4/S5 for serine, L-aspartic acid and L-valine shared the same route-mediated by FLS with dihydroxyacetone as the key intermediate. This schematic figure can be combined and shown in the maintext with the corresponding data (all the main findings, with Estimation of reaction Gibbs energy). There are many inconsistencies of enzyme names in the figures, eg. TPI vs Tpl. (a potential Figure 3)
3. Figure S8 will be sufficient, whereas Figure S6 and S7 can be deleted. I don't see the necessity of providing redundant information. Similarly, the results for L-glutamate and L-proline can be organized into one section since they have shared metabolic route. (a potential Figure 4)
4. Figure S11AB seems not necessary to me. They are essentially the same information to the metabolic routes as described in Figure S1-S8. The results of Figure S11C can be supplemented into different sections as mentioned above. The last section might focus on the scalability of the systems. (a potential Figure 5)
5. Figure 1 requires further changes to emphasize the modular design (intermediate/route dependent?). There is no point to highlight the alanine production in this figure. I could not directly see the plug-and-play modular feature of this work by simply looking at the figures. Why not simply use “dot” instead of hexagon? It is a bit confusing as it looks like a benzyl ring.
Minor issues:
1. There are still a number of enzyme name inconsistencies in the maintext and SI.
2. There are two serine pathways proposed in the SI, pls only show the route that was studied, to prevent any confusion.

Open Access This Peer Review File is licensed under a Creative Commons Attribution 4.0 International License, which permits use, sharing, adaptation, distribution and reproduction in any medium or format, as long as you give appropriate credit to the original author(s) and the source, provide a link to the Creative Commons license, and indicate if changes were made.
In cases where reviewers are anonymous, credit should be given to 'Anonymous Referee' and the source.
The images or other third party material in this Peer Review File are included in the article's Creative Commons license, unless indicated otherwise in a credit line to the material. If material is not included in the article's Creative Commons license and your intended use is not permitted by statutory regulation or exceeds the permitted use, you will need to obtain permission directly from the copyright holder.
To view a copy of this license, visit https://creativecommons.org/licenses/by/4.0/

REVIEWER COMMENTS

Reviewer #1 (Remarks to the Author):

This work presents the production of six amino acids from methanol or co2-derived methanol via a set of enzymatic pathways. The pathway design is great and involves several so-called plug-and-play modules. The synthesis of all six amino acids is demonstrated with high conversion yield, at least for some of them. Generally, it meets the novelty and the significance standard of the journal and can be published after major revisions.
-The manuscript format, writing typos, data presenting are suggested to be examined carefully and improved.
-The introduction is suggested to be further refined. Its current format reads like a report rather than a scientific paper.

We would like to thank the Reviewer for the detailed and constructive comments. In response, we have revised the introduction in both style and length, and we have shortened those sections that most closely resembled report-style paragraphs. As the introduction was substantially rewritten, it is difficult to indicate individual changes; the revisions concern lines 45–112.

-The hexagon in Fig 1 is kind of misleading. It looks like a phenyl group. It may be changed to cycle or sth else.

Thank you for this suggestion. From our perspective, the figure does not convey the presence of phenyl groups; therefore, we believe that modifying the representation of the carbon structures is not necessary. The figure is also explained in the accompanying caption.

-The Fig 2C and Fig 3 could be refined with more details or in another more-straightforward format. Fig 2C uses 10 mM C2 or C3 substrates reacted for 5 h, which Fig 3 uses 30 mM methanol for 24 h. In the text, those data are grouped based on the amino acid type and reported together. It is just kind of not very reader-friendly.

Thank you for this suggestion. We agree that presenting the approaches by amino acids in the text, while organizing the figures by approach, may be confusing for readers. To address this, we have revised the relevant sections of the manuscript and restructured the text to introduce the approaches first. The Figures have been reorganized accordingly: Figure 2 was transferred to the supplementary information. Figure 3 now follows the description of modules 1, 2 and 3, and Figure 4 follows the description of the complete system from CO_2. We hope that these changes improve clarity and readability.

-The data presenting could be further optimized. For example, the Fig S10 is abrupt. Why it only presents the alanine as the byproduct for the synthesis of proline? Why not showing other byproducts under other conditions? Indeed, as the production of many amino acids are demonstrated, the byproduct issue should be particularly discussed for those involved.

Thank you for this suggestion. We agree that presenting L-alanine as a byproduct, particularly only in the context of the proline cascade, may appear somewhat abrupt. Our intention was to highlight a potential underlying cause of this observation, namely the dual activity of LeuDH. Its higher activity toward pyruvate compared with α-ketoglutarate semialdehyde can lead to an imbalance between pyruvate and glycolaldehyde, which in turn may contribute to the reduced formation of L-proline.
In response to this comment, we have revised the manuscript accordingly. We now provide a more comprehensive discussion of byproduct formation, particularly with respect to L-alanine formation across different systems (lines 300 – 315, 375 – 380, 387 - 395). Furthermore, we have added metabolomics data on cascade intermediates for the upscaled reactions (381 – 389, 417 – 424 and SI).

-The legend in the Fig 4 seems not right. The color shown is not the same as the legend describes. Besides, in Fig 4B, why the DHA is not ~220 mM C at the point of 25 h ? It is only ~105 mM C based on the figure.

Thank you for this precise observation. The color coding in the text has been revised to match the colors used in the graph. In addition, the observed concentration difference results from the dilution step. To clarify this point, we have added a more detailed explanatory paragraph (lines 359 - 369) in the main text as well as in the figure caption.

-For valine production from CO2-derived methanol, as the data shown in Fig 4, why the conversion yield (37%) is much lower than those from methanol directly (78%)? Why the conversion almost stops at 12 h?

Thank you for this well-founded question. Because of the substantially higher initial methanol concentration (300 mM compared with 30 mM), we converted the one-pot system into a two-step process. This adjustment was necessary because downstream enzymes in modules 2 and 3 are sensitive to elevated formaldehyde concentrations, while FLS (the formaldehyde-converting enzyme) exhibits relatively low catalytic rates despite its stability.
Owing to the intrinsic conversion pathway from methanol to dihydroxyacetone, we first aimed to accumulate dihydroxyacetone and subsequently added the enzymes from modules 2 and 3 to convert it to L-valine. However, several limitations became apparent. First, the 300 mM methanol was not fully converted to dihydroxyacetone, which reduces the overall calculated yield of L-valine. Second, residual methanol may still impair enzyme activity or accelerate enzyme inactivation.
In addition, dihydroxyacetone itself is a reactive compound. Its carbonyl group can react with amines that are present at high concentrations in the reaction mixture (e.g., free ammonium, protein residues, or buffer components). Consistent with this, dihydroxyacetone was completely depleted after 12 hours, and L-valine production ceased at the same time. As no accumulation of pathway intermediates was observed, we assume that side reactions leading to unknown products occurred. Identifying these products would be an important next step, but this lies beyond the scope of the present proof-of-principle study.

-A table summarizing all the conversion yields of those amino acids is suggested. It can also contains the comparison with other technologies of in vitro amino acids synthesis from C1 sources, or highlights the advantages of the method used in this present work.

Thank you for the suggestion. In response, we have included a summary table in the revised manuscript to improve clarity and provide a concise overview of the relevant information (Table 1).

-Fig 5 is unnecessary. Please remove it.

In response to this comment, we have removed Figure 5 from the revised manuscript.

Reviewer #2 (Remarks to the Author):

This study presents a multi-enzyme cascade system design for synthesizing a series of amino acids from methanol, CO_2, and ammonia. Through modular replacement and enzymatic adjustments, the authors successfully achieved the synthesis of amino acids with carbon chain lengths ranging from C2 to C6. The work constitutes a notable achievement in demonstrating in vitro enzymatic synthesis of amino acids, offering a well-executed case study for modular biocatalytic systems. The systematic approach to pathway engineering and module optimization provides valuable insights for synthetic biology applications in value-added chemical production.
However, the data presented in the manuscript lack systematic organization, and the optimization process of the multi-enzyme catalytic system is described in an overly cursory manner. Critical scientific inquiries into the roles of key enzymes and pivotal catalytic steps remain insufficiently explored. Furthermore, the schematic depiction of the amino acid synthesis pathway lacks clarity in crucial mechanistic details. The authors are advised to comprehensively refine the manuscript by strengthening experimental rigor, elaborating on enzyme screening/engineering strategies, clarifying pathway design principles, and providing in-depth mechanistic discussions. The revised manuscript should be resubmitted for reevaluation after addressing these fundamental issues.
The authors are strongly advised to address the following critical aspects in their revision:

1.The step-by-step optimization workflow should be systematically elaborated, including enzyme ratio adjustment and screening of reaction conditions (pH, temperature, cofactor levels), to clarify how to maximize catalytic efficiency and pathway yield.

We would like to thank the Reviewer for the positive feedback on our work and for the helpful suggestions for revising the manuscript. We agree that the description of reaction conditions and enzyme selection in the main text was too brief. In response, we have added a new section outlining key considerations regarding enzyme selection and the required properties under the applied reaction conditions (lines 188 - 232). In addition, we have expanded the discussion of enzyme ratios and the rationale behind them (lines 234 – 256, 268 – 315).
As this study represents a proof of concept, it is possible that the optimal conditions for each individual pathway have not yet been fully established. However, the primary aim of this work was to develop a modular system that allows rapid exchange of pathway components while keeping most parameters constant. Achieving this required a compromise, and we therefore selected conditions that were compatible

with the majority of enzymes, even if this meant that the specific optima for some enzymes were not fully reached.
Our strategy for enzyme selection focused on previously characterized enzymes or those showing strong potential for compatibility within combined systems—for example, by sharing cofactors or metal requirements, or by exhibiting catalytic promiscuity that could be exploited across multiple cascades (e.g., LeuDH, which can be applied in different pathways).

2.Experimental data supporting the selection and engineering of pivotal enzymes (e.g., carbon chain elongation enzymes, ammonia assimilation enzymes) must be supplemented.

We have supplemented additional data on enzyme selection, including the activities and promiscuities of key enzymes involved in ammonia assimilation (lines 164 – 186) and Supplementary Figure S10). Furthermore, we have added a more detailed explanatory section in the main text regarding enzyme selection, covering FLS enzymes (lines 115 - 136).

3.The inhibitory effects of elevated methanol concentrations on amino acid carbon yield require systematic investigation. The authors are strongly advised to optimize the reaction system under high substrate-loading conditions to mitigate metabolic flux bottlenecks and enhance product titer.

Thank you for this suggestion. We agree that optimization under high substrate concentrations will be required to achieve industrially relevant titers. Methanol is known to exert inhibitory effects on several enzymes, and a detailed analysis of this effect could be valuable for further optimization.
In response, we have added metabolomics analyses of potential intermediate products in the L-valine and L-aspartic acid systems. No accumulation of intermediates was observed, leading us to conclude that side products are likely formed but have not yet been identified. These may arise from the reactivity of compounds such as dihydroxyacetone with amines. However, a comprehensive analysis of all potential byproducts is beyond the scope of this initial proof-of-concept study.

4.The methanol concentration-dependent alteration of the partitioning of the C2/C3 products of FLS-in particular the ratio of DHA to glycolaldehyde and its cascading effect on the glutamate/proline biosynthetic pathway remain mechanistically unresolved. This critical metabolic bifurcation node needs to be studied rigorously by performing.

Thank you for this suggestion. We agree that this is a very sensitive point. A previous study by Massad and Banta (https://doi.org/10.1002/bit.28217) demonstrated notable kinetic differences between formolase variants. We have made use of these extensive characterizations and selected the suggested enzyme variant for each specific purpose.

While a more detailed mechanistic analysis would indeed be valuable to understand how the enzyme catalyzes the formation of C2 or C3 molecules and why some variants terminate at C2, such an analysis falls beyond the scope of the present

manuscript and would be more appropriate for a dedicated mechanistic or characterization-focused publication.

Further enzyme engineering would be necessary to achieve either a one-to-one product ratio, or, conceptually, to split the system into three separate pots rather than two-steps, as done for valine and aspartic acid synthesis. In such a setup, methanol could be converted to glycolaldehyde in one pot using a specific enzyme (as exemplified for glycine), and to dihydroxyacetone in a second pot using a dedicated enzyme (as exemplified for L-valine). Both products could then be combined with modules 2 and 3 to achieve a one-to-one ratio, which should result in higher concentrations of L-glutamic acid and L-proline.

The use of the FLS “wildtype” enzyme in this manuscript served as a temporary solution. This enzyme lacks specificity for a single product and produces mixtures that are not in a one-to-one ratio. Its use here was intended to exemplify the possibilities of the system rather than to provide an optimized solution.

Reviewer #3 (Remarks to the Author):

Aminoacids are vital components of the food chain. Carbon dioxide is supposed to be the impornat source of C1 products in the future. Enzymatic catalysis and heterogeneous catalysis are known to have advantages and disadvantages, which may be the barriers for the potential commercialization. The paper is focused on the inventive combination of heterogeneous catalysis aiming at the production of methanol by CO2 hydrogenation and catalysis by enzymes as a downstream conversion of methanol produced at the first stage into valuable aminoacids. The authors presented this strategy by using not just one example of an aminoacid but the impressive range of amino acids with a different number of carbon atoms. Furtheremore, the authors tried to estimate the potential of this new technology for tow geographic regions and showed the applicability of the cascade technology. The paper seems to be a significant contribution to the fields of CO2 utilization and artificial food, especially produced from a waste. The following comments should be taken into account in the revision of the manuscript:
- The authors considered (and tested) a commercial catalyst, which provides a low efficiency in CO2 hydrogenation. Now new catalysts have been reported with a CO2 conversion reaching 25-30% and the optimal temperature of the process was found to be close to 250oC. Therefore, the authors may correct the figures to show a better performance of the cascade technology and improved economics/energetics.

Thank you for this suggestion. It is true that methanol synthesis from CO_2 is industrially conducted with higher CO_2 conversion and methanol yields. So, when envisioning industrial application of the introduced process cascade, an economically more beneficial performance of the step of methanol synthesis can be considered. However, the objectives of our experiments on CO_2-based methanol synthesis followed by enzymatic amino acid synthesis were a) to validate the approach of applying methanol derived from CO_2 (not from a CO-based syngas) as feed for the enzymatic process and b) to test the economically advantageous approach of applying the raw product mixture from methanol synthesis (without prior distillation) to the enzymatic process. For that purpose, operation of methanol synthesis at highest possible performance is not necessary. Moreover, the performance of methanol

synthesis is not only dependent on temperature, but also strongly on pressure. In terms of methanol yield, increasing temperature is even disadvantageous, as more CO is formed, lowering methanol yield. Unfortunately, in our reactor set-up we are limited to pressures of up to 40 bar. Therefore, under the applied conditions (limited by pressure) and with the utilized industrial catalyst, no better performance can be achieved.

Reviewer #4 (Remarks to the Author):

NCOMMS-25-29042-T

Plug and Play – Enzymatic Amino acid production from Methanol and Carbon dioxide
This study describes one-pot enzymatic systems for synthesizing serine, L-aspartic acid, L-valine, L-glutamic acid, and L- proline, form methanol, ammonia, and carbon dioxide. However, the reported titers for the abovementioned amino acids are relatively low ranging from 1-10 mM. The manuscript has enormous data, but it was poorly organized.
Major issue:
1. The reported titers for the amino acids are relatively low ranging from 1-10 mM. How is it compared to engineered cells to produce amino acids from methanol via metabolic engineering approaches? For instance, metabolic engineering of methanol-utilizing host such as Pichia pastoris will be a straightforward method, and it will simplify the enzyme purification process that will significantly reduce the preparation cost.

Thank you for these important comments. We acknowledge that the titers reported in our study are relatively low. To provide context, we have included data from methylotrophic hosts for amino acid production (line 82 – 88). Natural methylotrophs can achieve high titers for certain amino acids, such as glutamic acid and lysine, but the variety of amino acids produced remains limited. In engineered hosts that utilize methanol as the sole carbon source, amino acids are primarily consumed for the organism's own metabolism rather than being overproduced.
A major challenge in these systems is metabolic flexibility. While methylotrophs can produce mixtures of amino acids, fine-tuning the system to produce a single amino acid in high yield requires extensive metabolic engineering. In contrast, our modular system allows rapid switching to different amino acids simply by replacing one module, something not feasible in living organisms, where a new strain must be created for each target amino acid. Furthermore, the flexibility of our system enables cascades to be split into two- or three-step setups, allowing accumulation of intermediates with lower toxicity, such as dihydroxyacetone in the L-valine cascade. Increasing titers is a future goal, and enzyme engineering campaigns will be essential for this purpose. However, such optimization lies beyond the scope of this proof-of-concept study.
In regard of the suggestion to use *Pichia pastoris* for the production of enzymes, also for reducing the preparation costs we fully agree with the reviewer. This will be part of future work.

2. All the enzyme parts used in this study are previously reported. There is no gain-of-new-information, which leads to the poor novelty. The authors have previously

published a Chem Catalysis paper on alanine production from methanol. It seems to me that it is a simple expansion of the same design to several more amino acids.

Thank you for this comment. We respectfully disagree with this assessment. While L-alanine is indeed a simple amino acid that can be directly derived from pyruvate, focusing solely on this overlooks the novelty of our work. Although a subset of the individual reactions are newly demonstrated, the reductive amination of α-ketoglutarate semialdehyde represents a previously unreported activity of *bs*LeuDH. Similarly, the selective oxidation of glycolaldehyde to glycolate demonstrates a level of substrate specificity not previously described for this enzyme. Moreover, the individual product cascades themselves are challenging to implement, particularly when starting from C1-substrates or even CO_2, which further highlights the practical significance of our system.

More importantly, the central innovation of this study lies in the system-level design. Enzyme cascades are typically developed to produce a single, predefined target molecule. In contrast, we demonstrate a modular cascade architecture in which core components can be exchanged to deliberately redirect metabolic flux toward six different amino acids. This plug-and-play concept enables product diversification without redesigning the entire pathway. Furthermore, we combine known enzymes in non-natural configurations that explicitly exploit enzyme promiscuity and pathway engineering, creating functional biosynthetic routes that are difficult to achieve even for single products, and which do not exist in nature.
Together, these aspects establish the conceptual novelty of the work beyond the simplicity of any individual product and underscore the practical relevance of our approach in generating compounds that are otherwise difficult to obtain directly from CO_2.

3. The authors are encouraged to scale-up 1-2 amnio acid biosynthesis system, and provide more evidence that the enzymatic approach has advantage in turns of productivity and cost, compared to conventional engineered microbes via metabolic engineering. At this moment, all the reactions are only carried out at 100-200 µl. Can the authors demonstrate a 5-10 L reaction volume to provide scalability evidence?

Thank you for this suggestion. While amino acids can indeed be produced in high titers and volumes using microbial systems, our enzyme cascades serve as a proof of principle. To demonstrate preliminary scalability, we increased the substrate concentration from 30 mM to 300 mM, representing a tenfold increase. In parallel, the total reaction volume was expanded from 200 µL to 1 mL, corresponding to a 5-fold increase and a combined 50-fold scale-up in terms of substrate amount. To illustrate pure volume scaling, we further increased the reaction volume in the aspartic acid system from 200 µL to 15 mL, representing a 75-fold increase.
Scaling beyond these conditions is beyond the scope of this manuscript. Future work will focus on enzyme engineering to achieve technically relevant activities, followed by scaling to higher volumes, potentially up to the liter scale. We note that this approach is analogous to microbial systems, where strain engineering is typically performed prior to large-scale production.

4. If the biosynthetic pathways are not cofactor (NADPH, NADH, ATP) neutral, how is the cost of replenishing these cofactors?

Thank you for this comment. In the L-glutamate and L-proline systems, we incorporated an external cofactor recycling system using either NADH oxidase or formate dehydrogenase. All other cascades are intrinsically cofactor-neutral. Notably, a combination of the L-proline and L-glutamate pathways would also maintain cofactor neutrality.

5. Metabolite profiling should be provided during the course the reaction, to monitor the intermediate levels. These data should be provided in the SI.

Thank you for this suggestion. We have added metabolic profiling of intermediates for the two scaled cascade systems in the revised manuscript (lines 381 – 389 and lines 417 – 424 as well as Figure S12 and Table S9).

6. The figures in the maintext are poorly presented. Some of the biosynthetic routes can be organized in a better way, and combined with the corresponding data in the maintext.

Thank you for this suggestion. We have revised the figures and improved their integration into the main text.

7. Since the serine production is relatively low, it is recommended to move the data to the SI.

Thank you for this suggestion. Although the formation of serine is low, we consider it an important component of the overall system. For this reason, we have retained it in the main text.

Minor issues:
1. The enzymes and genes should be formatted in a uniform way. For instance, ItaE or ItAE.
2. All strains should be full name at the first appearance, and as abbreviated forms thereafter. For instance, line 450-460. (ATCC 7953) should not be an italicized form.
3. All restriction enzymes should have the first 3-letters in the italicized form (BsaI).
4. There are numerous grammar errors in the maintext and SI. Many of chemicals should be in the capital size.

Thank you for this suggestion. We appreciate your careful reading and have corrected the formatting and grammatical errors throughout the manuscript and SI.

Point-by-point response to the reviewers' comments

Reviewer #1 (Remarks to the Author):

The authors have addressed most of my previous concerns. However, the questions and suggestions from review 4 were not addressed well in my opinion.

Moreover, I feel that the “plug-and-play” of the multienzyme system was not well featured. For example, several enzymes should be grouped into one module, and several for the other. How these different modules could be organized and how the adaptation issue could be solved?

Also, it might be noticed that in these months, several works on cell-free multienzymatic synthesis of CO2-derived glycine and aspartate have come out (https://advanced.onlinelibrary.wiley.com/doi/10.1002/adsc.70238; https://pubs.acs.org/doi/10.1021/acssuschemeng.5c11185), which would hurt the novelty of this work..

We would like to thank the Reviewer for the interesting and constructive comments. In response, we have revised the manuscript. Most importantly, it now features a new Figure 1 introducing the Plug and Play multienzyme system. In particular, it illustrates the modular architecture by showing the individual modules as puzzle pieces that can be combined and exchanged differently to give rise to a diverse set of target amino acids. Whilst also showing the in-depth pathways (Figure 1b), it simultaneously draws a big picture to more easily grasp the idea of the modular design (Figure 1c) and different pot systems (Figure 1d). Related enzymes are now shown in the same color according to the module they belong to. We hope that this improves clarity.

Regarding the recently published works on glycine and aspartic acid transformations, one of the cited studies (glycine cascade) shows partial similarity in terms of the target transformation, however it employs a different set of enzymes and a distinct cascade implementation. The second study is based on a fundamentally different cascade architecture and reaction sequence. Importantly, in contrast to these examples, our work focuses on a modular Plug and Play cascade platform that enables systematic reconfiguration of enzymatic modules to access different product outcomes. In addition, we now refer to the mentioned references in the main text.

Reviewer #2 (Remarks to the Author):

NCOMMS-25-29042A

Plug and Play – Enzymatic Amino acid production from Methanol and Carbon dioxide
This study describes one-pot enzymatic systems for synthesizing serine, L-aspartic acid, L-valine, L-glutamic acid, and L-proline, form methanol, ammonia, and carbon dioxide. But the overall manuscript is still poorly organized after revision. It is difficult for non-expert to follow the logic. Although the metabolic routes for glycine and serine lack novelty, I found that the design of metabolic routes for L-glutamate and L-proline is very elegant and insightful. The manuscript might focus more on the key findings on L-glutamate and L-proline production from CO2. The revision partially addressed my concerns, but there are still more improvements required. I personally did not advocate using purified enzymes for amino acid synthesis as the cost of enzymes might be higher than the product itself. Nevertheless, this work might have some insights that would inspire others in the field.

Major issue:
1. As shown in Figure S1 and S2, these figures can be combined together since glycine and serine share the same pathway of glycolate as the intermediate. The combined metabolic route can be supplemented in the main text, followed by all the corresponding data to support (all the main findings, with Estimation of reaction Gibbs energy). By doing so, it will favor the readers to better understand the concept of “Plug and Play” and modular design. (a potential new Figure 2)
We would like to thank the Reviewer for the in-depth and constructive comments and appreciate your careful reading. We agree that presenting separate figures for amino acids sharing the same pathway is redundant and that transferring the combined metabolic routes from the SI to the main text and presenting these together with all the key findings including the thermodynamics data, the initial and optimized shortened cascades and the results of the complete cascades starting from methanol helps in following the logic and recognizing the modular design. In response, we have reorganized Figure 1 and Figure 2 accordingly: Figure 1 now introduces the modular architecture of our multienzyme system including the pathways of all six target amino acids. Herein, the color coding has been revised to match the colors used throughout the manuscript. Figure 2 illustrates the entirety of the theoretical data (i.e., the estimation of reaction Gibbs energy) and the experimental results (column-wise), structured according to individual groups of amino acids produced (row-wise, i.e., first two rows for glycine and serine). We opted for this kind of organization instead of creating individual figures for amino acids with combined routes, since we are convinced that this better aligns with the main text, which we have previously structured according to modules unfolding the system from the back, i.e., starting with the amino acid production module going *via* the cofactor regeneration module until the C1 fixation module. As we wanted to avoid crowding in Figure 2 and simultaneously saw the benefit of introducing the different cascades from the very beginning on, we are now showing a detailed overview of the six pathways in Figure 1b. To better relate the subsequent data to the metabolic routes, we have added the chemical structures of key intermediates in the graphs on the thermodynamics (Figure 2a-i). Thereby, we hope to be able to connect Figure 2 to Figure 1b and guide the reader through the individual pathways and the related data more smoothly.

2. Figure S3/S4/S5 for serine, L-aspartic acid and L-valine shared the same route-mediated by FLS with dihydroxyacetone as the key intermediate. This schematic figure can be combined and shown in the main text with the corresponding data (all the main findings, with Estimation of reaction Gibbs energy). There are many inconsistencies of enzyme names in the figures, e.g. TPI vs TpI. (a potential Figure 3)

Thank you for this suggestion. As we have transferred the pathways from the SI to the main text (Figure 1b), we have merged the routes for L-aspartic acid and L-valine in one that now only splits in two at the key intermediate phosphoenolpyruvic acid. This can now be seen in the middle branch of the pathway overview in Figure 1b. The corresponding data has now been gathered in Figure 2 with the middle rows being related to L-aspartic acid and L-valine, the two amino acids derived from dihydroxyacetone alone.

3. Figure S8 will be sufficient, whereas Figure S6 and S7 can be deleted. I don't see the necessity of providing redundant information. Similarly, the results for L-glutamate and L-proline can be organized into one section since they have shared metabolic route. (a potential Figure 4)
Thank you for this comment. We have removed the two additional figures and have fused Figure S8 with the major figure showing all pathways (i.e., Figure 1b, right branch). This is followed by all the corresponding data to demonstrate L-glutamic acid and L-proline production (Figure 2, bottom rows). Moreover, we appreciate your positive feedback on our design of the metabolic routes for L-glutamic acid and L-proline. To focus more on these precious compounds, we have indeed highlighted their routes from C1 building blocks exclusively and the corresponding data in our manuscript (lines 288–290 and 420–421).

4. Figure S11AB seems not necessary to me. They are essentially the same information to the metabolic routes as described in Figure S1-S8. The results of Figure S11C can be supplemented into different sections as mentioned above. The last section might focus on the scalability of the systems. (a potential Figure 5)
We would like to thank the Reviewer for these suggestions. To indeed avoid redundancy, we have eliminated Supplementary Figure 11A and 11B. The data previously presented in Supplementary Figure 11C has been ordered according to groups of amino acids (i.e., glycine and serine, L-aspartic acid and L-valine, L-proline and L-glutamic acid) and is now displayed in Figure 2j-o. In terms of scalability of our systems, we have effectively kept this data in the last section including Figure 3.

5. Figure 1 requires further changes to emphasize the modular design (intermediate/route dependent?). There is no point to highlight the alanine production in this figure. I could not directly see the plug-and-play modular feature of this work by simply looking at the figures. Why not simply use "dot" instead of hexagon? It is a bit confusing as it looks like a benzyl ring.
Thank you very much for sharing your perspective, which helped us in coming up with a new Figure 1 illustrating the modular design. Figure 1a has been kept deliberately simple, merely introducing the three different modules including the color coding. The puzzle pieces shown in one color represent the exchangeable modules which can be combined in different fashions with puzzle pieces from other modules. With Figure 1b demonstrating the detailed amino acid production pathways designed and evaluated in this study, we thought that Figure 1c could be helpful in seeing the big picture of the Plug and Play approach by narrowing the intricate metabolic routes down to the key compounds (i.e., substrate, branching points and products). Lastly, Figure 1d adds to this strategy by opening up for alternative systems going beyond one-pot reactions. We are no longer showing alanine production, as it is not directly dealt with in this work. As we have deleted elements and icons like hexagons, we hope to remove confusion with benzyl rings. Concluding, we hope that these changes improve clarity.

Minor issues:
1. There are still a number of enzyme name inconsistencies in the main text and SI.

Thank you for this precise observation. We have carefully gone through the nomenclature for the enzymes in our manuscript and have now named all enzymes uniformly, both in the main

text and SI. In response, we have also exchanged the enzyme names IlvC, IlvD, and LtaE for KARI, DHAD, and LTA, respectively, to conform to the names of all other enzymes.

2. There are two serine pathways proposed in the SI, pls only show the route that was studied, to prevent any confusion.

Thank you for this suggestion. We agree that presenting an alternative, second serine pathway can be confusing. We have thus removed this route from the SI. In line with your above suggestions, we have also combined the cascades for serine and glycine into one shared pathway given their common amino acid precursor glyoxylic acid. This is now shown in Figure 1 along with the pathways of the remaining amino acids.
